# Zwitterionic microgel preservation platform for circulating tumor cells in whole blood specimen

**DOI:** 10.1038/s41467-023-40668-1

**Published:** 2023-08-16

**Authors:** Yiming Ma, Jun Zhang, Yunqing Tian, Yihao Fu, Shu Tian, Qingsi Li, Jing Yang, Lei Zhang

**Affiliations:** 1 Department of Biochemical Engineering, School of Chemical Engineering and Technology, Frontier Science Center for Synthetic Biology and Key Laboratory of Systems Bioengineering (MOE), Tianjin University, Tianjin, 300350 China; 2 Department of Breast Cancer, Tianjin Medical University Cancer Institute and Hospital, Key Laboratory of Breast Cancer Prevention and Therapy, Key Laboratory of Cancer Prevention and Therapy, Tianjin’s Clinical Research Center for Cancer, National Clinical Research Center of Cancer, Tianjin Medical University Cancer Institute and Hospital, Tianjin, 300060 China

**Keywords:** Biomaterials - cells, Inhibitory RNA techniques, Biomaterials - cells, Biomedical engineering, Tumour biomarkers

## Abstract

The immediate processing of whole blood specimen is required in circulating tumor cell-based liquid biopsy. Reliable blood specimen stabilization towards preserving circulating tumor cells can enable more extensive geographic sharing for precise rare-cell technology, but remains challenging due to the fragility and rarity of circulating tumor cells. Herein, we establish a zwitterionic magnetic microgel platform to stabilize whole blood specimen for long-term hypothermic preservation of model circulating tumor cells. We show in a cohort study of 20 cancer patients that blood samples can be preserved for up to 7 days without compromising circulating tumor cell viability and RNA integrity, thereby doubling the viable preservation duration. We demonstrate that the 7-day microgel-preserved blood specimen is able to reliably detect cancer-specific transcripts, similar to fresh blood specimens, while there are up/down expression regulation of 1243 genes in model circulating tumor cells that are preserved by commercial protectant. Mechanistically, we find that the zwitterionic microgel assembly counters the cold-induced excessive reactive oxygen species and platelet activation, as well as extracellular matrix loss-induced cell anoikis, to prevent circulating tumor cell loss in the whole blood sample. The present work could prove useful for the development of blood-based noninvasive diagnostics.

## Abstract (web summary)

Blood specimen stabilization for the preservation of circulating tumor cells remains challenging. Here, the authors present a zwitterionic microgel platform for long-term hypothermic preservation of circulating tumor cells in the whole blood of cancer patients for noninvasive diagnostics.

## Introduction

The investigation of rare cells in the peripheral blood can provide important cellular or molecular information of the human body^1,2^. Recently, the advanced innovations in precise rare-cell technologies, e.g., circulating tumor cell (CTC)-based liquid biopsy, represent a breakthrough in clinical noninvasive diagnostics and cancer status monitoring^3,4^. The key insights of tumors, such as prognostic information, drug resistance mechanism, and metastasis development, can be comprehensively exploited by CTC-based liquid biopsy with high sensitivity (98% of breast cancers) and high specificity (99% of ovarian cancers) rates, benefiting patients for cancer therapy^5,6^.

However, the immediate process of whole blood specimen is required to insure the accurate molecular data for guiding clinical decision, while it needs to take a period of days for blood specimen shipment and waiting in line^7–9^. For example, there are only six commercially available centers for CTC detection (CellSearch) in the U.S., where the inspected specimens nationwide are sent^10^. Once removed from their native environment, most of CTCs in whole blood cells are degraded within 5 h, and shipment and handling of samples may even lead to total loss of CTCs owing to their fragility and rarity^11–13^. In a clinical study, the labeled tumor cells in whole blood have documented >60% loss within 5 h; meanwhile, significant RNA degradation in cells can be detected within 2–4 h^9^. Moreover, >83% of CTC losses are attributed mostly to cell apoptosis within 72 h, although the blood specimen is preserved using commercial protectants^4,14,15^.

Chemical fixatives are used to conventionally stabilize whole blood specimens, at the expense of sacrificing cellular activity and RNA integrity^9,16–19^. Alternatively, cryopreservation technology can enable long-term preservation of bio-samples, but highly concentrated cytotoxic cryoprotectants (40% glycerol or dimethyl sulfoxide), complex cryoprotectant removal procedures, and cryogenic conditions (−80 °C or −196 °C) are required^8,20,21^. Although several novel designed copolymers were used for efficient cell cryopreservation^22–25^, the freezing-thawing process can inflict damage in CTC viability and genotype, resulting in failure of quality control to profile CTCs^26–29^. Liquid protectant-based hypothermic preservation (HP) at 1 °C–35 °C (e.g., refrigerated temperature of 4 °C) is a current state-of-the-art shelf-storage method of whole blood specimens, due to its availability for clinical analysis and diagnostics^7,30,31^. However, few exceptions can hypothetically extend the viable preservation time toward preserving targeted rare cells in whole blood. The remaining studies focused on the addition of agents (e.g., caspase inhibitors, platelet activation inhibitors) into the liquid commercial protectants specific to blood, achieving an unfavorable protection efficacy^9,32–34^.

**Fig. 1:**
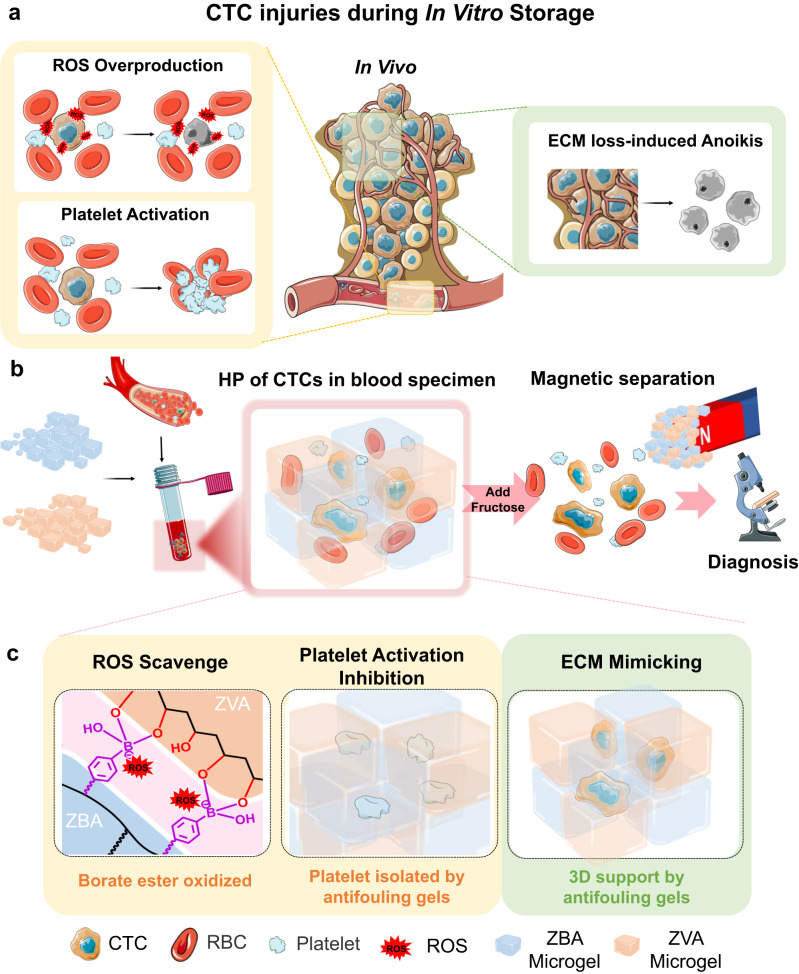
A Zwitterionic microgel preservation platform for CTCs in whole blood specimen. **a** The shedding process of CTCs from the in vivo tumor site (middle) and lesions of CTCs in whole blood specimen during HP (left and right). **b** The assembling process of magnetic microgels and ZBVA hydrogel formation for CTC preservation and effortless retrieval. **c** The mechanism of CTC protection against HP lesions by using the microgel platform.

Herein, we comprehensively investigated the main injuries of fragile CTCs in whole blood specimen, and its prolonged HP poses several challenges (Fig. 1a): 1) excessive reactive oxygen species (ROS) mainly induced by other cells, e.g., red blood cells (RBCs, 96% in whole blood)^35,36^; 2) cold-induced platelet activation leading to blood coagulation^37,38^; and 3) extracellular matrix (ECM) loss-induced anoikis of CTCs^39,40^. Because they have escaped from in vivo tumor tissue, in which ECM provides a 3D support to CTCs, into the flow of peripheral blood. Based on the clarified mechanism of CTC injuries, we established a zwitterionic magnetic microgel platform, which can prevent these injuries and enables stabilization of whole blood specimens for long-term HP, as well as effortless retrieval of targeted rare cells— CTCs, as described below (Fig. 1b, c).

## Results

### Construction of zwitterionic microgel preservation platform

**Fig. 2:**
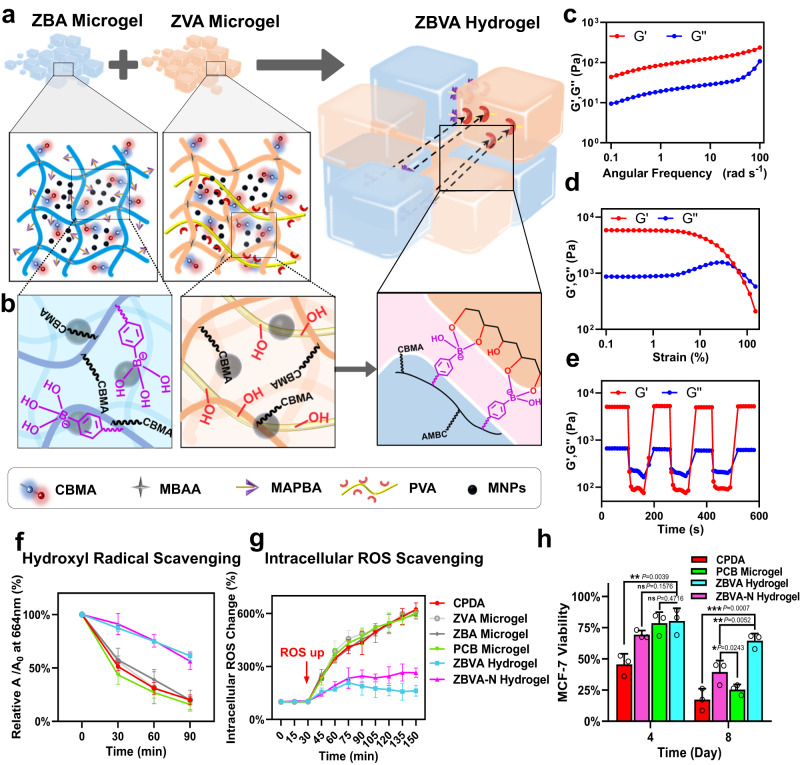
Formation and properties of the ZBVA hydrogel. **a** Schematic diagram of the ZBVA hydrogel formation by ZBA and ZVA microgel assembly. **b** The microstructure diagram of the two types of microgels and boronic ester bonds in ZBVA hydrogel. Rheological properties (**c**) of ZBVA hydrogel upon a constant strain of 1%, a strain between 0.1% and 100% (**d**), and step strain switch from 1% to 300% (**e**). The ROS scavenging performance (**f**) and intracellular ROS (**g**) of MCF-7 cells in CPDA or gels. **h** The viability of MCF-7 cells during different HP periods. For **f**–**h** values represent mean ± s.d. of *n* = 3 independent experiments and the two-tailed Student’s *t* test related *p* values are indicated.

Based on the mechanism analysis of the CTC lesions in whole blood specimen during HP, we designed a magnetic zwitterionic-phenylboronic acid (ZBA) and zwitterionic-polyvinyl alcohol (ZVA) microgel assembly to form a self-healing ZBVA hydrogel (Fig. 2a, b, and Supplementary Figs. 1 and 2). The microgel-based preservation platform is proposed to protect model CTCs against HP lesions, including ROS scavenging, inhibition of platelet activation, and ECM mimicking, as shown in Fig. 1c.

The ZBA microgels were Fe_3_O_4_ nanoparticle (MNP)-embedded single heterogeneous polymeric networks crosslinked with zwitterionic carboxylbetaine (CBMA) and methylacrylamide phenylboronic acid monomers. The ZVA microgels were designed as MNP-embedded interpenetrating polymeric networks based on PCBMA and polyvinyl alcohol (PVA). Approximately ~100 nm of MNPs were uniformly distributed in microgels (Supplementary Fig. 3), endowing them with the magnetic property. These magnetic microgels and their network structures were verified using the Fourier Transform Infrared Spectroscopy (Supplementary Fig. 4), Raman Spectroscopy (Supplementary Fig. 5), X-ray photoelectron spectroscopy (Supplementary Fig. 6), SEM mapping technologies (Supplementary Fig. 7a), tensile strength (Supplementary Fig. 7b), and differential scanning calorimetry tests (Supplementary Fig. 7c, d and Supplementary Table 1). The results show an interpenetrating polymer network of ZVA microgels. After complete equilibration, the equilibrium water contents of the microgels were 93% and 98%, respectively, indicating their superior swelling performance. Then, ZBA and ZVA microgels were obtained by repeatedly extruding bulk gels through the copper sieve until the size of microgels was slightly greater than that of normal cells (microgel size = ~50 μm in Supplementary Fig. 2).

The self-healing ZBVA hydrogel was built owing to the boronic ester formation between phenylboronic acids and diols on two types of microgels (Fig. 2a, b), and its chemical structure was demonstrated in Supplementary Figs. 5 and 6. The rapid assembling and self-healing process was firstly studied using different mass ratios of ZBA and ZVA microgels (Supplementary Fig. 8a). With the addition of PBS, either of the two types of microgels alone cannot form a stable hydrogel until their mixture with 1:1 of mass ratio. A stable ZBVA hydrogel could be rapidly constructed (~40 s), as shown in Supplementary Fig. 8b, and excessive PBS solution was unable to result in hydrogel collapse, owing to the presence of stable boronic ester bonds. Additionally, the hydrogels exhibited malleable and injectable performance (Supplementary Fig. 8c). The microscopic photographs in Supplementary Fig. 9a and Supplementary Movie 1 illustrate the assembling process of ZBA and ZVA microgels, as well as the ambient autonomous self-healing behavior of the resultant ZBVA hydrogel. In SEM images, the dense 3D polymeric networks in ZBVA hydrogel were verified (Supplementary Fig. 9b). The rheological results in Fig. 2c, d further demonstrate the elastic behaviors of ZBVA hydrogel and in Fig. 2e presents its self-healing property. Contrary to its stability in PBS, the ZBVA hydrogel quickly dissociated (~3 min) in a sugar solution that is compatible to cells, due to the interaction between boronic acid and 1,2-diol; then, the dissociated microgel assembly can be effortless enriched by magnetic separation (Supplementary Fig. 10). We further verified the anti-biofouling and cytocompatibility properties of the microgels, as shown in Supplementary Fig. 11. Overall, a preservation platform in terms of mild cell-encapsulation process, biocompatible material-based preservation, and effortless hydrogel separation was established.

### ROS scavenging, platelet stabilization and ECM-mimic support to cells by ZBVA hydrogels

Compared to rare CTCs, RBCs account for 96% of cells in the whole blood, and they are required to be protected in a reductive environment owing to the high oxygenated states. During HP, the by-product superoxide anions induce the chain reaction of oxidation in RBCs, mainly inducing the overproduction of ROS, such as hydrogen peroxides (H_2_O_2_) and hydroxyl radicals (•OH), in the microenvironment of the whole blood specimen^41,42^. For rare CTCs in the blood, RBC-produced ROS induces the peroxidation of membrane lipids and proteins, leading to cell apoptosis. Besides ROS overproduction from other cells, cold-induced platelet activation is another primary cause of the loss of CTCs during HP. The undesired self-aggregation of platelets can result in the damage of blood samples and apoptosis of CTCs. Although the traditional anticoagulation agents (e.g., sodium citrate) can chelate calcium ions and, thus, inhibit calcium-dependent platelet clotting, there are still other pathways and coagulation factors, such as thrombin and collagens, which can activate platelets^37,43^. The third proposed lesion to CTCs in specimen is the absence of ECM. CTCs escaped from in vivo tumor tissues, in which ECM provides both chemical interaction and physical support to cells. Once separated from ECM, the cytoskeleton disassembly of CTCs induces the activation of caspase proteases, leading to programmed cell death named as anoikis^44^ (Fig. 1a).

We established the microgel-based preservation platform that was proposed to protect model CTCs from the above-mentioned three types of lesions during HP at 4 °C (Fig. 1c). Firstly, the Transwell culture assay was used to investigate the effects of single variable, ROS scavenging or ECM-mimic support to cells, for prolonged HP periods of breast cancer cells. The following three preservation systems were designed and constructed: (1) cell-embedded ZBVA hydrogel system for both ROS scavenging and ECM mimicking (Supplementary Fig. 12a); (2) cell-embedded PCB microgel preservation system for 3D ECM-mimic support (Supplementary Fig. 12b); and (3) ZBVA-N hydrogel system, where ZBVA hydrogel was separated with cells by Transwell and solely for the purpose of ROS scavenging. (Supplementary Fig. 12c).

Firstly, the scavenging of ROS, including H_2_O_2_ and •OH, was evaluated in three preservation systems without cells. As shown in Supplementary Fig. 13a, in both ZBVA and ZBVA-N hydrogel preservation systems, 50% of H_2_O_2_ was decreased within 30 min and almost 100% was eliminated in 210 min. Contrarily, in the PCB microgel system, as well as the control group, i.e., CPDA (commercial protectant), there was a negligible decrease in H_2_O_2_ content. Figure 2f presents the ROS scavenging activity of these systems against •OH by using methylene blue (MB) as a probe. •OH, produced by Fenton reaction, can rapidly decrease the absorbance of MB solution in both the PCB microgel system and CPDA solution, while the decreases in ZBVA and ZBVA-N systems were significantly slowed down, indicating their •OH-scavenging activity (Fig. 2f and Supplementary Fig. 13b). To confirm the ROS scavenging activity for cells during HP, the breast cancer cells (MCF-7) were used in the three preservation systems. Figure 2g shows, initially, the negligible oxidative stress to cells in all the three systems owing to their excellent biocompatibility; until adding the ROS-up reagent, a large amount of ROS was immediately produced in each system. Compared to the continuous increase in the intracellular ROS level in the CPDA and PCB systems, the value of ROS in cells remains more stable after 90 min in the ZBVA and ZBVA-N systems after 90 min, attributable to the reduction of the boronated bonds. Additionally, the flow cytometry results (Supplementary Fig. 14a, b) and fluorescence microscopic images (Supplementary Fig. 14c) at the 150-min cell incubation in the systems further demonstrated that the ZBVA/ZBVA-N systems could efficiently eliminate the over-expressed ROS from cells.

Secondly, we utilized these preservation systems to investigate the effect of 3D ECM-mimic support for HP (at 4 °C) of cells. As shown in Fig. 2h and Supplementary Fig. 15, cell viability was inversely proportional to the preservation period in all these systems. At day 4, almost half of cell deaths occurred in the CPDA sample, the ZBVA and PCB systems still maintained >75% viability, while a slightly lower survival rate was observed in the ZBVA-N system. During 8–12 days, the ZBVA hydrogel system showed the significantly highest viability, and the higher rate observed in ZBVA-N than in the PCB system indicated that ROS overproduction was the primary cellular lesion in the late HP period. Cell survival rate of approximately 70% of in the ZBVA hydrogel on the 8^th^ day and of 50% on the 12^th^ day illustrated the critical role of synergistic protective effects (3D support and ROS scavenging) in long-term HP of cells. The 8-day preserved cells were found to sustain the cellular attachment ability, membrane integrity, and proliferation ability similar to fresh cells, indicating the normal functions of viable cells (Supplementary Fig. 16a, b). Supplementary Figure. 16c confirmed the activity of viable cells using the flow cytometry assays. Additionally, the well-preserved lung adenocarcinoma GLC-82 cells in the ZBVA system were also verified in Supplementary Fig. 17a–d. Moreover, we extruded zwitterionic hydrogels through different pore-diameter steel sieves to achieve different-size microgels (250 μm, 200 μm, and 100 μm) for preserving cells. The results showed non-gel formation by microgels with >250 μm diameter, while microgels with 200 or 100 μm were difficult to uniformly mix with living cells (10–20 μm), although cell preservation efficiency was negligibly affected by microgel sizes in Supplementary Fig. 18. Therefore, in consideration of operation and gel formation, we chose 50-μm-diameter microgels for the blood preservation platform.

**Fig. 3:**
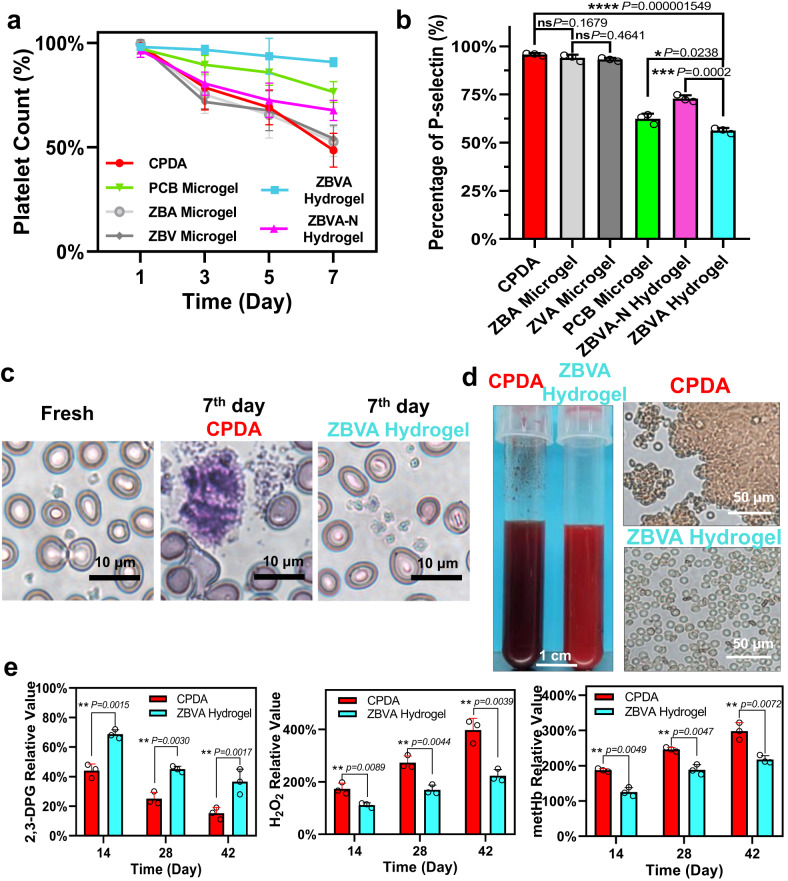
Inhibition of platelet activation in the ZBVA hydrogel system. **a** The platelet counts during the 7-day storage. **b** The platelet activation level on the 7^th^ day assessed by percentage of P-selectin by flow cytometry. **c** The representative images of fresh platelets (left), 7-day preserved platelets in the CPDA solution (middle) and ZBVA hydrogel (right). **d** The pictures and microscope graphics of the whole blood specimen preserved in the CPDA solution and ZBVA hydrogel. **e** The basic function of RBCs, including 2.3-DPG, H_2_O_2_, and metHb level, in 42-day preserved whole blood specimens. For **a**, **b**, and **e**, values represent mean ± s.d. of *n* = 3 independent experiments and the two-tailed Student’s *t* test related *p* values are indicated.

Thirdly, platelet activity and other cell viability in whole blood preservation were investigated, as shown in Fig. 3. There was almost a constant count (~94%) of the separated platelet rich plasma preserved in the ZBVA hydrogel, which was significantly higher than those of PCB and ZBVA-N systems, especially compared to 50% of the decrease in CPDA sample during the 7-day preservation (Fig. 3a and Supplementary Fig. 19a). The results demonstrated the efficient prevention of platelet disruption and aggregation by the ZBVA hydrogel system and indicated the requirement of both hydrophilic 3D isolated environment and ROS scavenging toward platelet stabilization. P-selectin (CD62) and free cytosolic calcium on the 7-day preserved platelets were tested to evaluate the activated platelets (Fig. 3b and Supplementary Fig. 19b, c). The activated platelets in the ZBVA system decreased by 36-50% compared to that in CPDA sample. Subsequently, the whole blood specimens of the patients were further preserved, shown in a schematic process in Supplementary Fig. 20, and the platelets and other cells in the specimens were investigated (Fig. 3c–e). The 7-day preserved blood samples were stained using Wright–Giemsa assay, and the graphics presents platelet aggregation in the CPDA group, whereas significant inhibition was observed in the ZBVA system (Fig. 3c). Additionally, the hematology analysis of other cell types in blood specimens, including white blood cells (WBCs), monocytes (MONs), lymphocytes (LYMs), granulocytes (GRAs), RBCs, as well as the indicators of RBCs, suggested a negligible decrease in the count of other cells and RBC functions after a 7-day preservation, compared with the fresh specimens. (Supplementary Fig. 21). To test the long-term cell stabilization and further verify the protection mechanisms of ZBVA hydrogel, we prolonged the preservation time to 42 days, which beyond the maximum shelf life of RBCs in CPDA for transfusion (35 days). As shown in Fig. 3d, the coagulation cascade was activated in one of the 20 blood specimens (coagulation problem happened in one sample among 20 samples) preserved in CPDA on the 10^th^ day, although the anticoagulant heparin was added. The abnormal blood clotting phenomenon in the blood specimens of tumor patients, so-called disseminated intravascular coagulation, is due to the abnormal coagulation pathways mediated by cancer procoagulant, tumor cell surface tissue factor, ROS, or other molecules/enzymes from the tumor environment^45,46^. Contrarily, the ZBVA systems can inhibit platelet activation relying on platelet isolation and ROS scavenging. At 42 days, the colors in all CPDA-preserved blood samples darkened (more Hb was oxidized to metHb) while ZBVA presented a bright red color (less oxidized metHb) (Fig. 3d and Supplementary Fig. 22a). The results of hemolysis, oxygen-binding capacity (2,3-DPG), and oxidation process (level of metHb and H_2_O_2_) fully demonstrated the whole blood stabilization and anti-oxidant property of the ZBVA system (Fig. 3e and Supplementary Fig. 22b, c).

Overall, we can conclude the well protection of CTCs in whole blood from hypothermic lesions, including ROS overproduction, cold-induced platelet activation and ECM loss-induced anoikis, highly dependent on the unique properties of ZBVA hydrogel system. The boronated bonds of ZBVA result in the reduction of ROS; meanwhile, 3D zwitterionic surroundings not only provide ECM-mimic support to cells but also mitigate excessive ROS production, as previously reported^47^. The antifouling zwitterionic 3D matrix can isolate the platelets and provide a super-hydrophilic environment to inhibit their activation, owing to the specific zwitterionic structure with equal positive and negative charges^32^ (Fig. 1c).

### Whole blood stabilization toward CTC preservation

**Fig. 4:**
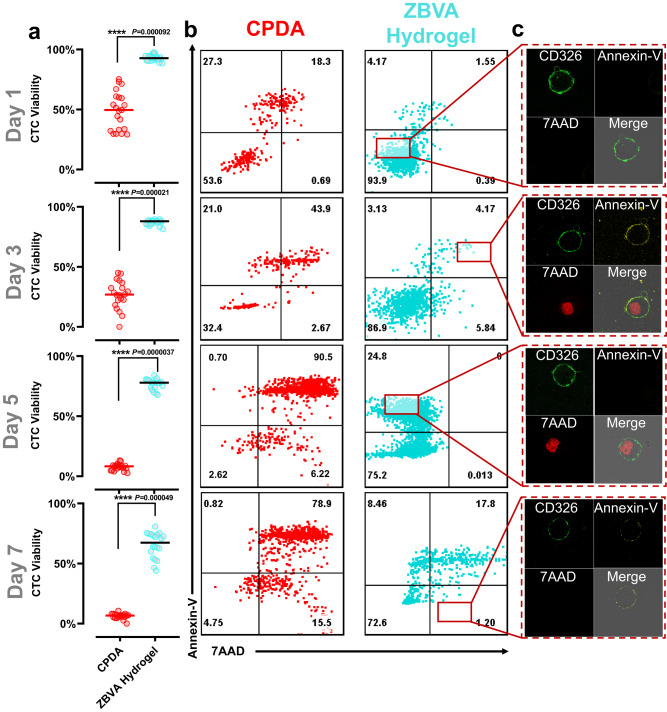
Model CTC life span to 7 days in the whole blood specimen of 20 patients. **a** The viability of CTCs in patient blood preserved by CPDA and ZBVA hydrogel during 7 days. The apoptosis and live/death state of CTCs in the whole blood tested by flow cytometry (**b**) and confocal fluorescence microscopy (**c**). For **a**, values represent mean ± s.d. of *n* = 20 independent experiments and the two-tailed Student’s *t* test related *p* values are indicated.

Based on the systematic investigations of the ZBVA hydrogel against three types of lesions, a cohort study of blood specimens of 20 breast cancer patients was evaluated, as shown in Fig. 4. Owing to the rareness of CTCs in the whole blood sample (1 of CTCs per 10^9^ hematologic cells in the blood of metastatic cancer patients), only a small fraction of CTCs (~5 cells in 1 mL of patient blood sample) would result in the large randomness and poor repeatability on cell viability during long-term preservation^48–50^. To address it, we developed a model system by spiking cancer cells into patient blood samples to accurately evaluate the performance of preservation platform^30,31,51^. We performed a 7-day preservation of model CTCs in whole blood specimens by ZBVA and CPDA as a control. Upon magnetic separation, blood samples were processed using a CTC enrichment kit (Supplementary Fig. 23a). The isolated model CTC-rich solution was subsequently investigated by fluorescence confocal microscopy, and the results are shown in Supplementary Fig. 23b. Obviously, both CD45^+^ and CD326^+^ cells could be detected in the fluorescence photographs, indicating that there were WBCs and CTCs in the enriched solution. The analysis from the blood analyzer presented the trace of RBCs, WBCs, and platelets in the enriched solution, achieving the detection thresholds for flow cytometry (Supplementary Fig. 23c). Then, flow cytometry was combined with rigorous gating methods to precisely distinguish apoptosis and death of the targeted model CTCs (Supplementary Fig. 24).

Only during the 1-day preservation, the viability of model CTCs in the CPDA solution was sharply decreased to 50% (Fig. 4a). As the FACS files in Fig. 4b and Supplementary Figs. 25 and 26 the process of CTC apoptosis and death showed that half of the cells initiated the apoptosis procedure on the first day, as detected with Annexin-V (Fig. 4c). More cells showed apoptosis and then quickly died within 2 days. On the 5^th^ day, all of the CPDA-preserved cells died. It can be speculated that anoikis primarily induced remarkable apoptosis, and subsequently ROS accumulation accelerated the CTC death process. Instead, in the ZBVA hydrogel system, almost none of CTCs initiated the apoptosis process during the first 3 days; only 25% of CTCs were found to have an apoptosis signal after the 5-day preservation. On the 7^th^ day, the mean viability of CTCs in 20 different blood samples was maintained at ~65%, doubling the viable preservation duration reported so far.

Table 1 concluded the characteristics of 20 donors, including age, blood type, cancer stage, and the post-preserved model CTC viability in their blood samples. The correlation analysis of recipients’ basic information and cell viabilities were calculated using SPSS software, and the results are shown in Supplementary Fig. 27. There was no correlation between patient characteristics and ZBVA-gel-preserved CTC survival in their whole blood. Supplementary Fig. 28 shows a heatmap presenting the preservation efficacy of CTCs in the blood specimens of 20 patients, suggesting that the preservation efficiency of ZBVA hydrogel was superior to standard commercial CPDA protectant. These results highlighted the versatility of the ZBVA hydrogel system toward long-term HP of targeted rare cells in whole blood specimens.

**Table 1:** Recipient characteristics and model CTC viability in their blood specimens preserved for 7 days

Recipient characteristics					Model CTC viability							
	Age	Blood type	Cancer stage		ZBVA hydrogel				CPDA			
					Day1	Day3	Day5	Day7	Day1	Day3	Day5	Day7
*Patient-1*	58	B	IIIA		91%	87%	79%	73%	34%	22%	13%	6%
*Patient-2*	61	A	IIA		92%	88%	78%	75%	42%	15%	7%	8%
*Patient-3*	47	B	0		94%	88%	81%	64%	30%	32%	10%	7%
*Patient-4*	68	O	IIIA		92%	86%	75%	54%	33%	39%	8%	8%
*Patient-5*	58	B	IIA		97%	81%	78%	76%	30%	30%	9%	6%
*Patient-6*	46	A	IIA		97%	84%	69%	71%	32%	27%	9%	7%
*Patient-7*	39	AB	IIB		94%	83%	74%	52%	75%	26%	13%	4%
*Patient-8*	43	O	IA		98%	89%	84%	74%	73%	32%	9%	8%
*Patient-9*	52	AB	IB		93%	89%	68%	81%	67%	23%	9%	6%
*Patient-10*	68	AB	IIA		95%	85%	78%	64%	29%	18%	4%	7%
*Patient-11*	68	A	IIIB		94%	89%	80%	63%	71%	45%	4%	7%
*Patient-12*	54	O	IIIA		95%	90%	81%	60%	30%	44%	3%	11%
*Patient-13*	36	B	IIA		94%	90%	80%	46%	54%	9%	5%	7%
*Patient-14*	44	O	IA		90%	88%	81%	44%	50%	40%	5%	6%
*Patient-15*	39	B	IIIB		91%	87%	79%	53%	59%	13%	9%	5%
*Patient-16*	38	A	IA		93%	88%	70%	63%	54%	29%	7%	7%
*Patient-17*	35	AB	IIB		88%	88%	78%	73%	60%	37%	8%	5%
*Patient-18*	46	O	IIA		91%	86%	72%	74%	51%	24%	7%	0%
*Patient-19*	44	B	0		89%	88%	74%	75%	61%	27%	8%	8%
*Patient-20*	62	B	IIA		92%	87%	78%	72%	44%	0%	9%	3%

All patients are female.

The informed consent for the publication of basic information (age, sex, blood type and cancer stage) in this research from all patients were provided.

### Molecular profiling of 7-day preserved model CTCs

The ultimate purpose of liquid biopsy is to discover the predictive biomarkers, the mechanism of drug resistance, and the efficacy of the personalized drug, relying on molecular profiling information in CTCs, such as the transcriptomic profiling of RNA. However, RNA is a fragile bioinformatics molecule in CTCs. RNA degradation will be directly induced by oxidative bursts and hemolysis in whole blood specimens^52^. As reported, a significant RNA degradation was observed within 4 h in ex-vivo blood^9^. Therefore, the World Health Organization has recommended the limitation of 2-h storage duration of extracted blood for precise RNA analysis^9,53^.

**Fig. 5:**
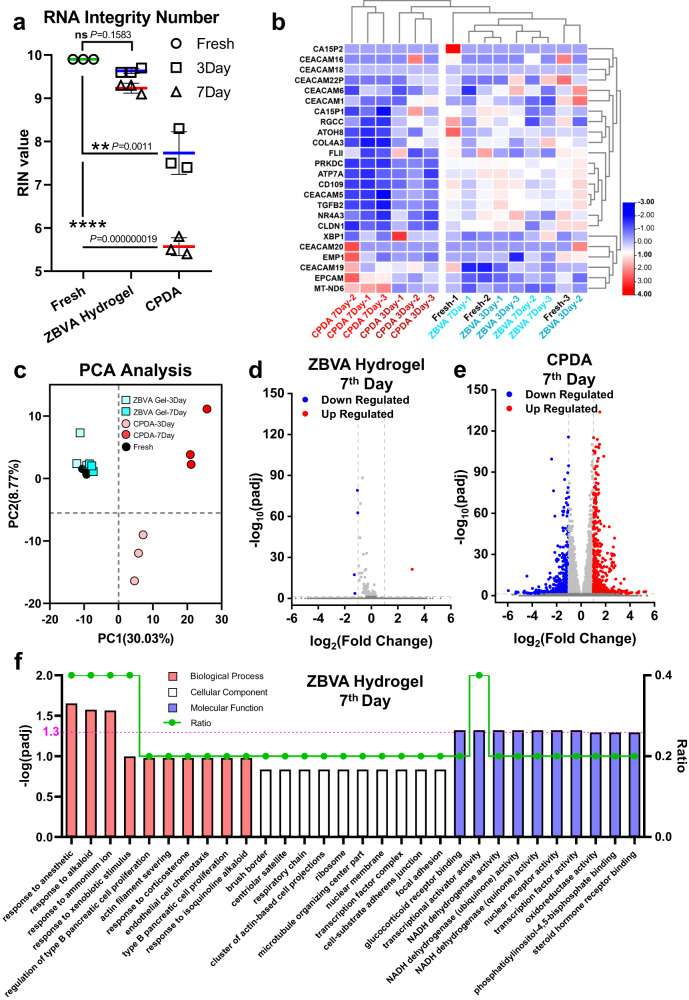
Model CTCs for molecular profiling including RNA sequencing and Gene Ontology (GO) enrichment. **a** The RNA integrity number (RIN) values of model CTCs isolated from fresh or preserved blood specimens. **b** Scaled heatmap of specific breast cancer gene expression in the model CTCs from the fresh or preserved blood specimens. **c** Principal component analysis (PCA) of CTCs from the fresh or preserved blood specimens. Significant gene expression changes in model CTCs from the ZBVA hydrogel preservation system (**d**) and CPDA solution (**e**). **f** Top ten significant changes in biological processes (BP), cellular component (CC), and molecular function (MF) in CTCs preserved in the ZBVA hydrogel on the 7^th^ day. The y axis on the left indicates the significance score shown as -log(padj). The right y axis indicates the ratio of the number of GO pathway correlated genes that map the total significant expressed genes (green line). The pink dashed line indicates where the -log(padj) equals 1.3, which is the boundary score between significance and non-significance. For **a**, values represent mean ± s.d. of *n* = 3 independent experiments and the two-tailed Student’s *t* test related *p* values are indicated.

Here, we validated the maintenance of RNA integrity in viable CTCs preserved with the ZBVA hydrogel system and CPDA for 7 days. The RNA quality (RNA integrity number, RIN) in the CTCs was firstly evaluated (Fig. 5a and Supplementary Fig. 29a). The mean RIN value of CTCs in ZBVA hydrogel was 9.63 on the 3rd day and 9.23 on the 7^th^ day, indicating a negligible difference from CTCs in the fresh blood samples. In stark contrast, CTCs retrieved from the CPDA solution had a greater amount of degraded RNA (RIN = 7.73 on the 3^rd^ day and 5.56 on the 7^th^ day). We further investigated the gene expression and signal markers on the preserved CTCs. The RNA-Seq, a whole-transcriptomic profiling approach, was used. Gene expression of the marker of breast cancer (CA and CEA gene family), CTCs (EPCAM and EMP), self-antioxidant system (MT-ND6), and proliferation (e.g., RGCC, ATOH8, and PRKDC) were detected, as shown in Fig. 5b. For the control group (CPDA), significant changes were found in almost all gene expressions, as compared with the fresh sample group. While there was no difference in the 24 genes in CTCs between the fresh blood sample and the sample preserved by using the ZBVA hydrogel. The principal component analysis (PCA) of all gene expression data in the 15 groups was applied to determine any significantly different orientations between the fresh and CPDA-preserved CTCs (Fig. 5c). The volcano plots in Fig. 5d–e presented only five gene changes in ZBVA-preserved CTCs, and they were all not cancer-specific genes. Instead, in CPDA-preserved CTCs, 1243 gene changes containing 646 gene expression upregulations and 597 gene downregulations were observed (Supplementary Fig. 29b). The top 30 up- and downregulated genes are listed in Supplementary Fig. 29c. We then carried out a gene ontology (GO) enrichment analysis of the biological process, cellular component, and molecular function terms (GO: BP, GO: CC, and GO: MF) for the up- and downregulated gene sets. The top ten significant changes in each term in the ZBVA system were selected and shown in Fig. 5f and those in the CPDA are shown in Supplementary Fig. 30. Strikingly, the changed terms of CTCs in the ZBVA system were only associated with the self-antioxidant systems, and the related genes were downregulated. Meanwhile, the genes in the same terms of CPDA-preserved CTCs were all upregulated, indicating the overexpression of self-antioxidant-related proteins or enzymes in cells. This phenomenon was attributed to the ROS accumulation in the CPDA, while ROS scavenging property of the ZBVA hydrogel. The other top ten significantly changed terms in the CPDA-preserved CTCs were all associated with the behavior of cell localization. We believe that the localization-related gene upregulation was due to ECM loss in the liquid CPDA-based environment.

In summary, the molecule profiling analysis reflected the high RNA quality and negligible gene changes in model CTCs preserved in the ZBVA hydrogel, which was attributable to the protective mechanism from the main HP injuries of the designed preservation system.

## Discussion

Herein, we designed a zwitterionic microgel-based preservation platform against three types of HP lesions toward rare CTCs in the whole blood specimen, including ROS overproduction, ECM loss, and platelet activation. It was attributed to the unique design of this microgel-based platform, which are as follows: the boronated bonds among microgel assemblies, and 3D support environment provided by the non-fouling polymeric network. Meanwhile, the MNPs in the microparticles can ensure effortless retrieval of preserved cells. In a cohort study of 20 cancer patients, this platform was verified to significantly prolong the lifespan of model CTCs in specimens to 7 days, which was twice as long as the current viable preservation duration. Moreover, the systematic molecule profiling analysis reflected the high RNA quality and negligible gene changes in CTCs preserved in this platform, compared to the up/down regulation expression of 1243 genes in the CPDA-preserved CTCs. This work clarifies the main lesions to the rare targeted cells in blood specimens, and the preservation platform reported here can pave the road to the development of blood-based noninvasive diagnostic systems.

## Methods

### Ethic approval statement

The Medical Ethic Committee of Tianjin Medical University Cancer Institute and Hospital conducted a serious rapid review of the mentioned research project. After review, the Medical Ethics Committee approved the research protocol (protocol number is bc2021066) and agreed to the study “Zwitterionic Microgel Preservation Platform for Circulating Tumor Cells in Whole Blood Specimen” for research publication. Human Samples and Data usage in this research follow Privacy Protection Principle, which effectively protect personal privacy.

### Materials

The carboxybetaine methacrylate (CBMA) monomer and magnetic Fe_3_O_4_ nanoparticle (MNP) were synthesized using a method previously reported^54^. Methylacrylamide phenylboric acid (MPBA) monomer, fructose, and polyvinyl alcohol (PVA) were procured from Heowns. Ammonium persulfate (APS), N,N’-Methylenebisacrylamide (MBAA), and N,N,N’,N’-Tetramethylethylenediamine (TEMED) were purchased from Acros. The MTT Assay kit was sourced from Abcam, while the CTCs EasySep kit was obtained from StemCell. Ltd. RPMI-1640 Memorial Institute-1640, DMEM Dulbecco’s Modified Eagle’s Medium, and FBS fetal bovine serum were all obtained from Gibco. DMSO and PBS phosphate-buffered saline were procured from Beijing Solarbio Science and Technology Company Co., Ltd. The Wright–Giemsa Stain Assay kit was sourced from Nanjing Jiancheng Bioengineering Institute. The Live/Dead Assay kit, HSA/FITC, and BSA/FITC were all purchased from Invitrogen. The Nicotinamide adenine dinucleotide (NAD^+^/NADH) analysis kit, hydrogen peroxide (H_2_O_2_) analysis kit, reactive oxygen species (ROS) analysis kit, and Fluo-4 AM calcium assay kit were procured from Beyotime Biotechnology Co., Ltd. CPDA was purchased from Shanghai Xinfan Biology and Science Company Co., Ltd, and the CPDA solution was prepared by mixing CPDA and deionized water (DI) water at a ratio of 1.5:10. The GLC-82 and MCF-7 cell lines were purchased from Meisen Cell Technology Company Co., Ltd, and the catalog numbers are CTCC-007-0001 and CTCC-0342-Luc2, respectively. Whole blood samples utilized in this study were donated by human breast cancer patients, the informed consent were provided.

The Annexin-V/7AAD apoptosis detection kit, CD326(EpCAM) monoclonal antibody-Alexa Fluor 488, CD45 monoclonal antibody-APC, and CD62P monoclonal antibody-PE were procured from Shanghai Universal Biotech Company Co., Ltd. The detailed information of each antibodies is described in Supplementary Table 2.

### Terminology of different gels in this work

ZBA, ZVA, and PCB hydrogels after the first synthetic step were named as ‘bulk ZBAZVA/PCB hydrogel’; after extruding steps, they were named as ‘ZBA/ZVA/PCB microgel’. ZBA and ZVA microgels could construct a ‘ZBVA hydrogel’.

### Cell preparation

#### Preparation of cancer cells

GLC-82 and MCF-7 cells were cultured in RPMI 1640 and DMEM medium containing 10% FBS and 1% P/S, respectively, at 37 °C in a cell incubator with 5% CO_2_. The cells were detached using trypsin-EDTA, collected by centrifugation at 114 g for 4 min, and finally resuspended in culture medium for further use.

#### Preparation of blood cells and platelets

Whole blood samples utilized in this study were from human donors. The red blood cells (RBCs) and platelets were enriched via density gradient centrifugation (114 g, 10 min) in SepMate tubes.

### Synthesis and characterization of bulk ZBA, ZVA, and PCB hydrogels

#### Synthesis of MNPs

Magnetic Fe_3_O_4_ nanoparticles (MNPs) were synthesized via a previously reported method^55^. In brief, 4.9 g FeCl_3_ 6H_2_O and 2.1 g FeCl_2_ 4HO were dispersed in 30 mL of DI water under nitrogen gas protection. Subsequently, 8 mL of strong ammonia was added to the solution under vigorous mechanical stirring, and the reaction temperature was gradually increased to 75 °C for 1 h. Finally, the MNPs were collected and purified using a magnet wash process with DI for three cycles. Dynamic light scattering (DLS) technology was used for size distribution detection of MNPs.

Synthesis of CBMA monomer: Briefly, 300 mL anhydrous acetone and 60 mL DMAEMA were added to a flask after the oxygen removing process. Then the mixture was stirred at room temperature until they were completely dissolved. Under continuous stirring at 0 °C, a solution of beta-propiolactone (21.82 mL, 0.35 mol) was carefully added dropwise to the flask. The reaction proceeded for a duration of 2 h. Following this, the resulting product was subjected to washing with ether and subsequently dried under vacuum conditions for a period of 24 h.

#### Synthesis of bulk ZBA hydrogel

The bulk ZBA hydrogel was produced using thermo-polymerization method reported before^56^. Briefly, 1.05 g CBMA and 0.525 g MPBA monomers were dissolved in 1.5 mL DMSO and 1.5 mL DI water to prepare the mixed solution. Then 0.3 g MNPs, 11 mg MBAA, 10 mg APS, 10 μL TEMED were added into the mixed solution and stirred for 5 min. The resulting solution was then transferred into a mold and heated at 60 °C for 6 h to obtain the bulk magnetic hydrogel.

#### Synthesis of bulk ZVA hydrogel

To prepare the PVA solution, solid 0.5 g PVA was dissolved uniformly in 5 mL DI water. Then, 2 g CBMA, 0.5 g MNPs, 20 mg MBAA, 20 mg APS, and 20 μL TEMED were added to the PVA solution and stirred for 10 min. The solution was then placed into a mold and heated at 60 °C for 6 h to create the first network of CBMA via one-step polymerization. The mold was subsequently transferred to a refrigerator at 4 °C for 8 h and then warmed to room temperature for 2 h. This process led to the successful synthesis of bulk ZVA hydrogel after the formation of the PVA network.

#### Synthesis of bulk PCB hydrogel

Bulk PCB hydrogel was prepared by the previously reported method^57^. Briefly, 1 g CBMA, 8 mg APS, 10 mg MBAA, 7 μL TEMED was dissolved into 2.5 mL DI water uniformly. Then the solution was placed at 60°C for 6 h to get the bulk hydrogel.

#### Thermogravimetry (TGA) test of various gels

The TGA system of METTLER-TOLEDO was used to perform the TGA test. An empty crucible was used to obtain a baseline measurement. The hydrogel sample was then placed in the crucible, purged with nitrogen, and heated at a rate of 10 °C/min from room temperature (25 °C) to 800 °C. The mass change data of the sample was recorded throughout the heating process.

#### Differential scanning calorimetry (DSC) test of various gels

Different hydrogel samples (5–10 mg) were placed in aluminum pans. For testing the T_g_ point, samples were cooled from 20 °C to −20 °C at a rate of 10 °C min^−1^, kept at −20 °C for 5 min. Then, samples were warmed at a rate of −5 °C min^−1^ to 200 °C. To detect the melting point, the samples (5 mg) were heated from 20 °C to 300 °C at a scanning rate of 5 °C/min.

#### X-ray photoelectron spectroscopy (XPS) test of gels

Three types of bulk hydrogel samples were freeze-dried before the test. The surface elements of the samples were analyzed by K-Alpha+ x-ray photoelectron spectrometer (Thermo Fisher, America) which recorded spectra within a range of 0–1400 eV at intervals of 0.1 eV.

#### Mechanical strength tests of gels

The mechanical properties of bulk hydrogels were tested by using a universal mechanical test machine. Specifically, a sample with a cross-sectional area of 5 mm^2^ and a length of 20 mm was subjected to a stretch rate of 5 mm min^−1^ at room temperature. The resulting tensile curves were used to calculate the elastic modulus of different samples.

#### Equilibrium water content (EWC) of bulk hydrogels

The hydrogel disks with 5 mm diameter were made from bulk hydrogels. Then, the hydrogel disks were allowed to swell to equilibrium state in DI water for 120 h. The swollen disks were weighed and dehydrated by freeze-drying for 72 h. The dried disks were also weighed.

The equilibrium water content of each hydrogel was measured by Eq. (1)

1 $${{{{{\rm{EWC}}}}}}=\left(1-\frac{{{{{{{\rm{M}}}}}}}_{{{{{{\rm{d}}}}}}}}{{{{{{{\rm{M}}}}}}}_{{{{{{\rm{s}}}}}}}}\right)\times 100\%$$

where M_s_ and M_d_ are the mass of swollen and dried hydrogels, respectively.

#### Protein adsorption test

Fluorescence-labeled BSA-FITC and HAS-FITC proteins were employed to investigate the protein adsorption of hydrogels. To this end, hydrogel disks were placed in 24-well plates, and 200 μL of protein solution was added to each well. After 2 h of incubation in the dark, the samples were rinsed at least 3 times and observed under a fluorescence microscope.

#### Cell adhesion test

In this study, the cell adhesion of hydrogels was evaluated using GLC-82, MCF-7, and human platelet cell lines. Specifically, 5 × 10^5^ cells/mL were seeded onto the hydrogel disks in each well and incubated at 37 °C under 5% CO_2_ for 72 h. After washing the hydrogels three times, the number of adherent cells was calculated by microscopy. The number of cells in a tissue culture polystyrene dish was used as a positive control (P, 100%). The original cell numbers (O) of the experimental groups were recorded, and the final values (F) were calculated using the following Eq. (2).

2 $${{\mbox{F}}}=\frac{{{{{{\rm{O}}}}}}}{{{{{{\rm{P}}}}}}}\times 100\%$$

#### Cytocompatibility test

The hydrogels were soaked in moderate culture medium for the extractions in culture chamber for 24 h. Then GLC-82 and MCF-7 cells with the concentration of 10^6^ cells/mL were added into 48-well plate with different extraction. After 72 h incubation, the live and dead cells were photographed in fluorescence microscope by using a Live/Dead Assay kit. The number of cells in tissue culture polystyrene dish was considered as the positive control (P, 100%). The original cell numbers (O) of experimental groups were determined, and the final values (F) were calculated using the following Eq. (3).

3 $${{{{{\rm{F}}}}}}=\frac{{{{{{\rm{O}}}}}}}{{{{{{\rm{P}}}}}}}\times 100\%$$

### Synthesis of ZBA, ZVA, PCB microgels

Microgels were prepared by extruding bulk hydrogels through copper wire meshes with varying mesh hole diameters, ranging from 500 μm to 50 μm. To achieve the desired size of ~50 μm, three types of bulk hydrogels were passed through each mesh at least twice. The microgels with diameters of approximately 200 μm and 100 μm were obtained during this process. All batches were then sterilized by ultraviolet for 48 h and lyophilized before the final use.

### Establishment and characterization of ZBVA hydrogel

#### Establishment of ZBVA hydrogel

Different mass ratio of ZBA and ZVA microgels with total mass of 60 mg were put into 1 mL CPDA solution. Then the bulk ZBVA hydrogels would be formed in solution within a short time.

#### Rheological characterization of ZBVA hydrogel

Rheological measurements were conducted using an MCR 302 rheometer. Oscillatory frequency sweeps with a constant strain of 1%, oscillatory strain sweeps with strains ranging from 0.1% to 100%, and step-strain test with step-strains ranging from 1% to 300% were all performed at room temperature.

#### Magnetic separation of microgels

The ZBVA hydrogel was rapidly dissociated within 3 min by using 3 mL 0.2 M fructose solution. The dissociated hydrogel could then be collected efficiently using a magnetic grate. Meanwhile, 3 mL of PBS solution were added to CPDA/blood samples or other experiment groups to maintain the volume consistency. The final dilution of different groups was maintained at the same level (Quadruple dilution).

#### In vitro hydroxyl radical (•OH) scavenging ability

To evaluate the •OH scavenging ability of different hydrogels, methylene blue (MB) was used for detecting the change of •OH concentration in various systems. Typically, MB (10 μg/mL) was added into •OH containing solution (mixture of 1 mM H_2_O_2_ and 0.2 mg/mL FeCl_2_ 4H_2_O) and incubated with different gels and CPDA solution. The absorption of whole system at 664 nm was measured every 30 min.

#### In vitro H_2_O_2_ scavenging ability

To evaluate the H_2_O_2_ scavenging ability, 1 mM H_2_O_2_ solution was incubated with different hydrogels or CPDA solution. The concentration of H_2_O_2_ was tested by H_2_O_2_ analysis kit every 30 min.

#### ROS scavenging ability in cell system

GLC-82 cells were plated at a density of 1 × 105 cells/mL in a 96-well plate and incubated for 24 h. Subsequently, 50 μL of DCFH-DA culture medium was added to each well and incubated for 1 h. The culture medium was then replaced with different hydrogels or CPDA solution, followed by the addition of 100 μL of ROS-up regent to each system. The fluorescence intensity was measured every 60 min using a microplate reader and fluorescence microscope.

### Characterization of cells after hypothermic preservation

#### Cell hypothermic preservation

Flow tubes, tube holders and all the other apparatus used in cell experiments were sterilized using an autoclave before use. 1 ×10^6^ cancer cells (1 ×10^9^ platelets or 1 ×10^11^ RBCs) and different lyophilized hydrogels was added into 1 mL CPDA solutions, and cells in CPDA solution was regarded as the control. ZBVA hydrogel system was established by adding 33 mg of ZBA and ZVA microgels into 1 mL CPDA solution with either 1 ×10^6^ tumor cells, 1 ×10^9^ platelets, or 1 ×10^11^ RBCs. All preservation systems were then straightly stored in a refrigerator (4 °C).

#### Whole blood specimen preservation

Whole blood specimens were mixed with CPDA at a ratio of 10:1.5 to obtain CPDA-preserved whole blood. To establish the ZBVA hydrogel system, 33 mg of ZBA and ZVA microgels were added into 1 mL of CPDA-preserved whole blood. All preservation systems were then immediately stored in a refrigerator at 4 °C.

#### Cell viability test

Cell viability after preservation was assessed using a Live/Dead Assay kit. Briefly, cell suspensions (20 μL) from various preservation systems were mixed with staining solution (80 μL) in a 96-well plate. The plate was then incubated at room temperature for 30 min in the dark. Fluorescence microscopy was used to capture images of the plate, and the number of live (green) and dead (red) cells was counted to calculate cell viability.

#### Cell attachment test

Following the preservation process, cells from various preservation systems were washed with PBS and resuspended in culture medium. The suspension was then seeded in 24-well plates and incubated for 12 h in a cell chamber. Different fields were chosen under the microscope and the number of cells attached in each field was quantified.

#### Cell proliferation test

The cells resuspended from different preservation systems were cultured in a 12-well plate, and the number of cells attached to the substrate was counted every 24 h to evaluate the proliferation ability of the cells.

#### Platelets counting

Platelet counts in various preservation systems were determined using a BM21B blood analyzer (Baolingman, China). The initial platelet counts were considered as the positive control (P, 100%). The original platelet counts (O) of experimental groups were measured and the final values (F) were calculated using Eq. (4).

4 $${{{{{\rm{F}}}}}}=\frac{{{{{{\rm{O}}}}}}}{{{{{{\rm{P}}}}}}}\times 100\%$$

#### Platelet p-selectin test

Platelets harvested from different systems were then resuspended at the concentration of 5 ×10^6^/mL in PBS. After centrifugation at 114 g for 8 min, concentrated platelets were stained with CD62P-PE gently homogenized in a vortex mixer. Platelets were then incubated at room temperature for about 30 min and analyzed by FASCAria III, the flow cytometer (BD Bioscience, USA). The data of fresh platelets were regarded as the negative control (N, 0% of P-selectin, no activated platelets). The data of platelets activated by appropriated collagen were regarded as the positive control (P, 100% of P-selectin, fully activated platelets)^58,59^. After testing the original data (O) of experimental groups, all final value (F) was calculated by Eq. (5).

5 $${{{{{\rm{F}}}}}}=\frac{4\times {{{{{\rm{O}}}}}}-{{{{{\rm{N}}}}}}}{{{{{{\rm{P}}}}}}}\times 100\%$$

The used platelets in control and experimental groups were from the same patient.

#### Platelet free calcium test

Fresh and preserved platelets were resuspended at the concentration of 5 ×10^6^/mL in PBS. After centrifugation at 114 g for 8 min, the concentrated platelets were stained by a calcium assay kit. Briefly, 500 μL 2 μmol/L of Fluo-4 AM was added in the concentrated platelets, and incubated in 37 °C of environment for 30 min. After the complete probe loading and washing by 3 times of PBS solution, the fluorescence intensity of platelets were detected by a microplate reader (Tecan M2000pro, Switzerland).

#### Whole blood staining method

Wright’s-Giemsa Staining method (Wright’s-Giemsa Staining kit) was used for whole blood staining.

#### Number changes of cells in whole blood

The number of different types of cells in whole blood was calculated by BM21B blood analyzer (Baolingman, China).

#### RBCs hemolysis test

RBCs hemolysis test. To prepare the standard curve of hemolysis, 40 μL aliquots of fresh RBC solutions were mixed with 960 μL of PBS (negative control) and DI water (positive control), respectively. The 100% hemolysis group was subsequently diluted to create additional hemolysis control groups at 90%, 80%, 70%…10% using DI water. Supernatants from each group were added to a 96-well plate and their absorbance at 450 nm was measured by a microplate reader. The constructed hemolysis standard curve is shown in the following Eq. (6).

6 $${{{{{\rm{Hemolysis}}}}}}=\frac{{{{{{\rm{OD}}}}}}\, {{{{{\rm{value}}}}}}-4.238}{2.32}\times 100\%$$

To test the hemolysis data of different experimental groups, 40 μL of RBCs solution from each group was added to 960 μL of PBS and the collected supernatant was measured at 450 nm. The hemolysis of RBCs in each experimental group was then determined by comparing the measured absorbance to the standard curve.

The used RBCs in control and experimental groups were from the same patient.

#### The oxygen-binding capacity, and oxidation level test of RBCs

Oxygen-binding capacity and the level of oxidation process of RBCs were evaluated by a 2,3-DPG test kit, H_2_O_2_ test kit, and metHb test kit. The data of fresh blood cells are regarded as positive control (P, represented 100%). After testing the original data (O) of experimental groups, all final value (F) was calculated by Eq. (7).

7 $${{{{{\rm{F}}}}}}=\frac{4\times {{{{{\rm{O}}}}}}-{{{{{\rm{N}}}}}}}{{{{{{\rm{P}}}}}}}\times 100\%$$

The used RBCs in control and experimental groups were from the same patient.

### Characterization of model CTCs after hypothermic preservation

#### Preparation of model CTCs

2000 of MCF-7 cells were added in 1 mL whole blood to simulate CTCs. Blood samples were extracted from different breast cancer patients.

#### Hypothermic preservation of whole blood with model CTCs

To preserve model CTCs in whole blood samples, ZBVA hydrogel system was established by adding 33 mg of ZBA and ZVA microgels into whole blood samples. The CPDA solution system was obtained by mixing 1 mL of blood sample with 150 μL of CPDA. All preservation systems were then straightly stored in a refrigerator (4°C).

#### Viability and apoptosis test of model CTCs

After separation from ZBVA and CPDA system, model CTCs in the whole blood were then enriched by CTCs EasySep kit with magnetic activated cell sorting method. To accurately detect model CTC viability and distinguish them from white blood cells, residual red blood cells, and platelets, the enriched solutions were stained with CD45-APC, Annexin-V-FITC, and 7AAD, and gently homogenized in a vortex mixer. After a 30-min incubation at room temperature, the stained solution was analyzed using the FASCAria III flow cytometer (BD Bioscience, USA). The results from Flow Cytometry tests were analyzed by FlowJo (version 10.6.2). The viability data of fresh model CTCs were considered as the positive control (P, almost 100%). The original data (O) of experimental groups were then tested, and all final values (F) were calculated using Eq. (8).

8 $$F=\frac{4\times {{{{{\rm{O}}}}}}-{{{{{\rm{N}}}}}}}{{{{{{\rm{P}}}}}}}\times 100\%$$

The used model CTCs in control and experimental groups were from the same specimen.

### Molecular profiling analysis of model CTCs after hypothermic preservation

#### RNA extraction and qualification

Model CTCs in ZBVA hydrogel and CPDA solution were enriched every 2 days during hypothermic preservation. Fresh model CTCs was recognized as the control. The separated model CTCs were used for molecular profiling analysis. RNA integrity was assessed by using RNA Nano 6000 Assay kit of the Bioanalyzer 2100 system (Agilent Technologies, USA).

#### RNA-seq expression

Total RNA was used as input material for the RNA sample preparation. Then, mRNA was purified by magnetic beads and broken into short fragments. The RNA sequencing libraries were constructed and sequenced by Qubit 2.0 Fluorometer and Agilent 2100 bioanalyzer. The different libraries were qualified the effective concentration by qRT-PCR, and pooled for Illumina sequencing.

#### RNA-seq data analysis

Clean data (clean reads) were obtained by removing irrelevant reads (containing adapter, poly-N). Then, FPKM of each gene was calculated as the gene expression level. Afterward, differential expression analysis of different groups was performed by DESeq 2 and gene with an adjusted *P*-value ≤ 0.05 was labeled as differentially expressed. The mapped reads of each sample were assembled by StringTie (version 1.3.3b) (Mihaela Pertea.et al. 2015) in a reference-based approach. Gene Ontology (GO) enrichment analysis was mapped and calculated by the clusterProfile R. GO terms (*P*-value ≤ 0.05) were significantly enriched by the differentially express genes.

The detailed information of reagents and instruments used in this section can be found in Supplementary Data 1.

### Statistical analysis

All data were presented as means and standard deviation (Means ± s.d.). To test the significance of differences, a paired two-tailed Student’s *t* test using Graph Pad was determined, as indicated in the figure legends. The Pearson and Spearman correlation coefficients were used in correlation analysis by SPSS software.

### Reporting summary

Further information on research design is available in the Nature Portfolio Reporting Summary linked to this article.

### Supplementary information

Supplementary Information

Description of additional supplementary files

Supplementary Data 1

Supplementary Movie 1

Reporting Summary

### Source data

Source Data

## Data Availability

The authors declare that the data that supports the findings of this manuscript can be found in the Supplementary Information and are available free of charge or available from the corresponding author upon request. Source data are provided with this paper. RNA-seq Data files are released publicly in the NCIB SRA repository. The Biosample accession codes and links are: “SRR25208120”, “SRR25208117”, “SRR25208114”, “SRR25208119”, “SRR25208116”, “SRR25208113”, “SRR25208121”, “SRR25208118”, “SRR25208115”, “SRR25208111”, “SRR25208108”, “SRR25208110”, “SRR25208107”, “SRR25208112” and “SRR25208109”. Any additional requests for information can be directed to, and will be fulfilled by, the lead contact. Source data are provided with this paper.
