# Supplementary material for: Zwitterionic microgel preservation platform for circulating tumor cells in whole blood specimen
Source: Nat Commun. 2023 Aug 16;14:4958. doi: 10.1038/s41467-023-40668-1 (PMC10432405; doi:10.1038/s41467-023-40668-1)
Supplement: Supplementary file 1 — Supplementary Information [file 41467_2023_40668_MOESM1_ESM.pdf]

Supplementary Information

A Zwitterionic Microgel Preservation Platform for Circulating Tumor Cells in Whole Blood Specimen

Yiming Ma[1,#], Jun Zhang[2,#], Yunqing Tian[1], Yihao Fu[1], Shu Tian[1], Qingsi Li[1], Jing Yang[1,], Lei Zhang[1,*]*

[1]Department of Biochemical Engineering, School of Chemical Engineering and Technology, Frontier Science Center for Synthetic Biology and Key Laboratory of Systems Bioengineering (MOE), Tianjin University, Tianjin 300350, China

[2]Department of Breast Cancer, Tianjin Medical University Cancer Institute and Hospital, Key Laboratory of Breast Cancer Prevention and Therapy, Key Laboratory of Cancer Prevention and Therapy, Tianjin's Clinical Research Center for Cancer, National Clinical Research Center of Cancer, Tianjin Medical University Cancer Institute and Hospital, Tianjin, 300060, China

Author Information

**Corresponding Authors: Jing Yang and Lei Zhang*

Email address: jing_yang@tju.edu.cn, lei_zhang@tju.edu.cn.

ORCID: Jing Yang: 0000-0002-2529-3947, Lei Zhang: 0000-0003-3638-6219

Author Contributions: Yiming Ma and Jun Zhang contributed equally to this work. All authors have given approval to the final version of the manuscript.

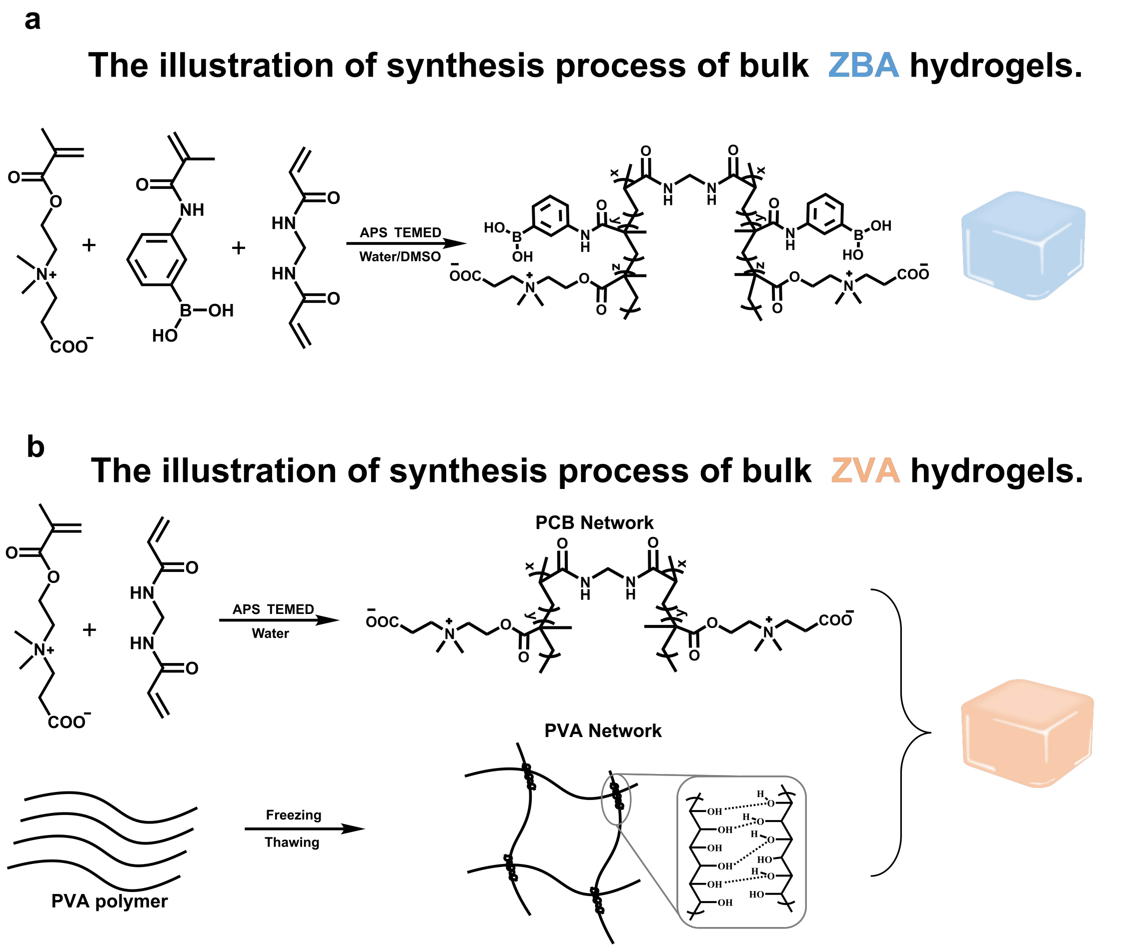

Supplementary Figure 1. The illustration of synthesis process of bulk (a) ZBA and (b) ZVA hydrogels.

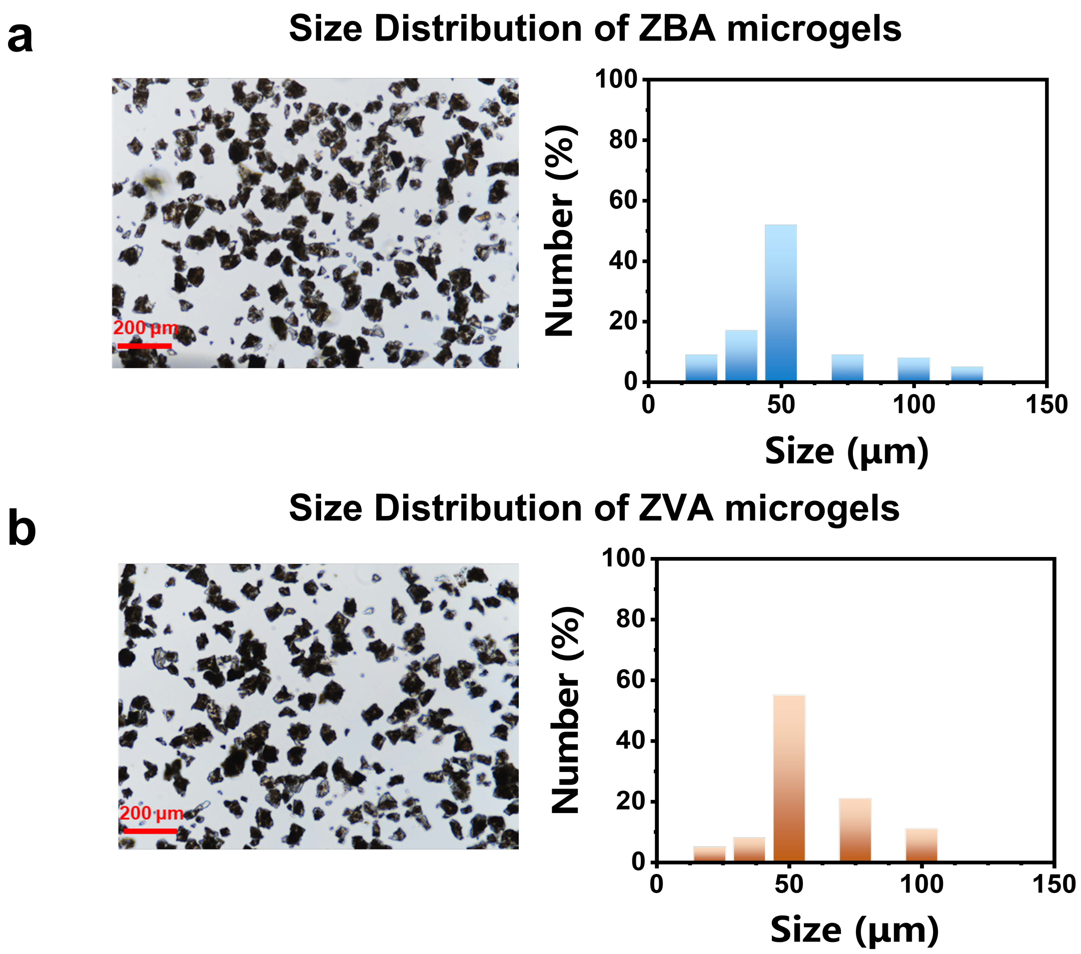

Supplementary Figure 2. Shape images and size distribution of (a) ZBA and (b) ZVA microgels.

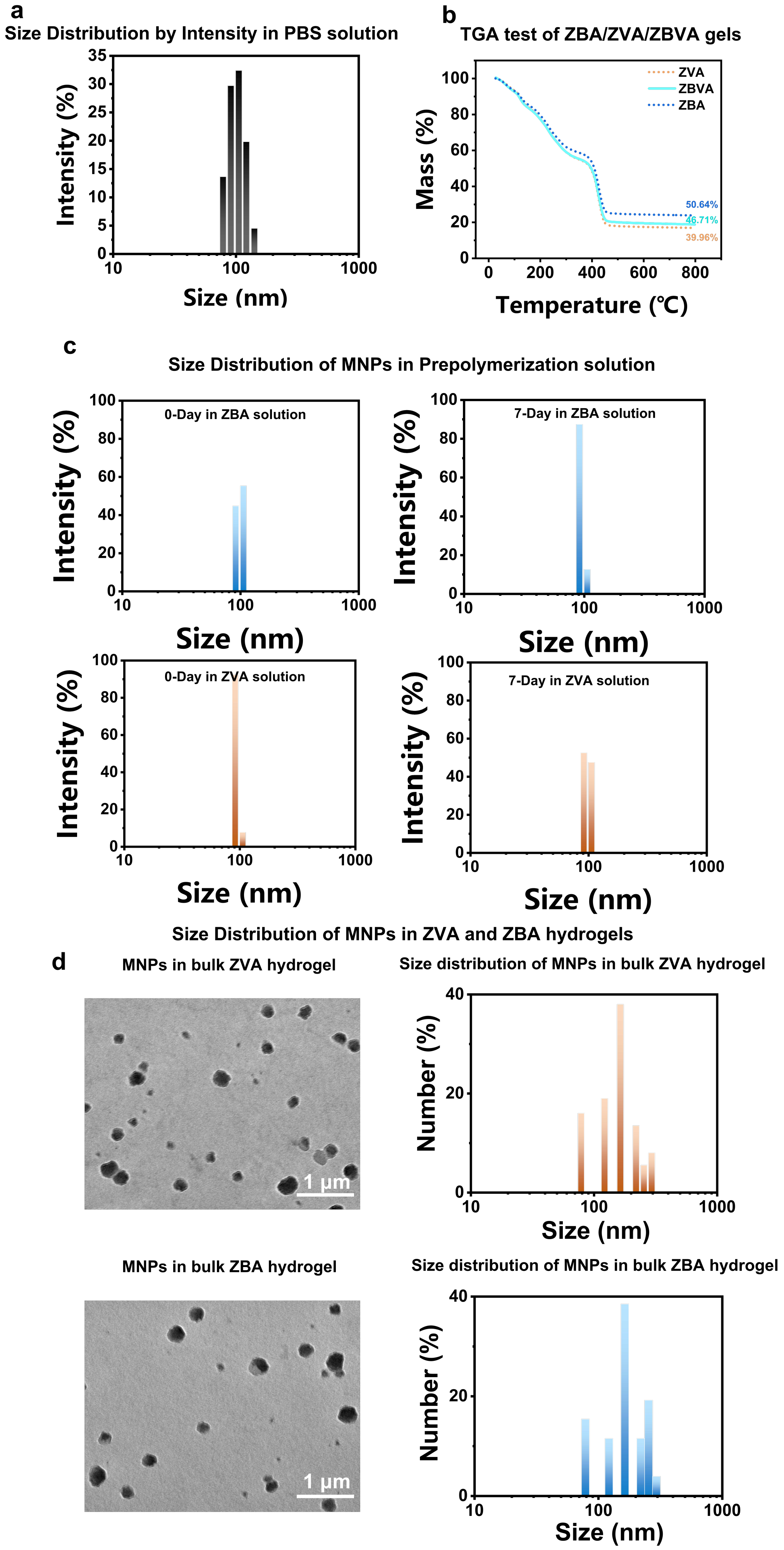
a
Size Distribution by Intensity in PBS solution
Intensity (%)
Size (nm)
b
TGA test of ZBA/ZVA/ZBVA gels
ZVA
ZBVA
ZBA
50.64%
46.71%
39.96%
Mass (%)
Temperature (℃)
c
Size Distribution of MNPs in Prepolymerization solution
0-Day in ZBA solution
7-Day in ZBA solution
0-Day in ZVA solution
7-Day in ZVA solution
Intensity (%)
Size (nm)
Size Distribution of MNPs in ZVA and ZBA hydrogels
d
MNPs in bulk ZVA hydrogel
Size distribution of MNPs in bulk ZVA hydrogel
1 μm
Number (%)
Size (nm)
MNPs in bulk ZBA hydrogel
Size distribution of MNPs in bulk ZBA hydrogel
1 μm
Number (%)
Size (nm)

Supplementary Figure 3. Size distribution and homodispersion of MNPs in ZBA and ZVA gels. a The size distribution of MNPs tested by DSL in PBS solution. The DLS test have shown that the size of MNPs is around 100 nm. **b** The TGA test of various bulk hydrogels. These gels exhibit a sharp reduction in weight between 100°C and 500°C, and the stabilized mass present the loading capacity of MNPs: 16.97% in the ZVA, 23.86% in the ZBA, and 18.86% in the ZBVA. **c** MNPs retain the stability in the ZBA/ZVA prepolymerization solutions for up to 7 days without clustering or sedimentation. **d** The homo-distribution of MNPs in the ZBA and ZVA microgels characterized by Cryo-TEM (Titan Krios 300 kV D3172, Thermo Fisher). The particle sizes of MNPs were 100-200 nm and uniformly dispersed in the microgels (~50 μm). It indicated that there was cluster formation at nanoscale in microgels, while uniform distribution of MNPs at microscale can endow ZVA/ZBA microgels with prompt magnetic separation property.

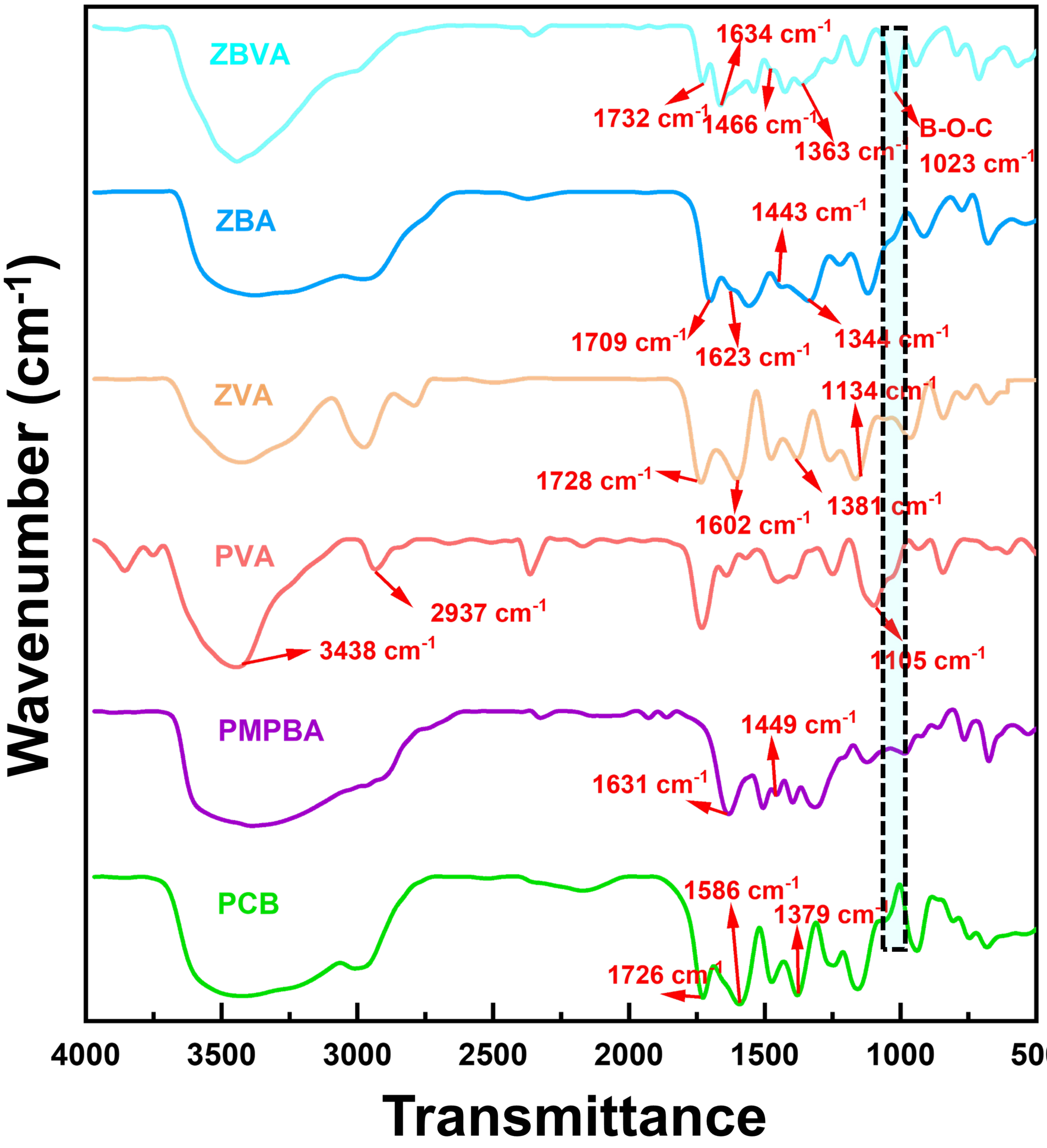

Supplementary Figure 4. The FTIR test results and characteristic peaks of different hydrogels. The FTIR spectrum of PCB hydrogel displayed characteristic peaks at 1726 cm^{-1} (C=O stretching), 1568 cm^{-1} (COO^- asymmetric stretching), and 1379 cm^{-1} (COO^- symmetric stretching)[1]. The spectrum of PMPBA hydrogel exhibited peaks at 1631cm^{-1} (C=C in benzene ring stretching) and 1449 cm^{-1} (B-O stretching)[2-4]. The spectrum of PVA hydrogel exhibited peaks at 3438 cm^{-1} (O-H stretching), 2937 cm^{-1} (C-H stretching), and 1105 cm^{-1} (C-O stretching)[5,6]. The characteristic peaks in the FTIR spectra of ZVA, ZBA, ZBVA hydrogels demonstrated their successful preparation. The spectrum of ZVA hydrogel displayed peaks at 1728 cm^{-1} (C=O stretching), 1602 cm^{-1} (COO^- asymmetric stretching), 1381 cm^{-1} (COO^- symmetric stretching), and 1134

cm cm^{-1} (C-O stretching). The spectrum of ZBA hydrogel exhibited peaks at 1709 cm^{-1} (C=O stretching), 1623 cm^{-1} (C=C in benzene ring stretching), 1443 cm^{-1} (B-O stretching), and 1344 cm^{-1} (COO^- symmetric stretching). In ZBVA hydrogel, the presence of an alcoholic hydroxyl groups and phenyboronic acid groups led to the formation of boronic ester bonds (B-O-C, characteristic peak at 1023 cm^{-1})[4,7]. It confirmed the successful preparation of ZBVA hydrogel.

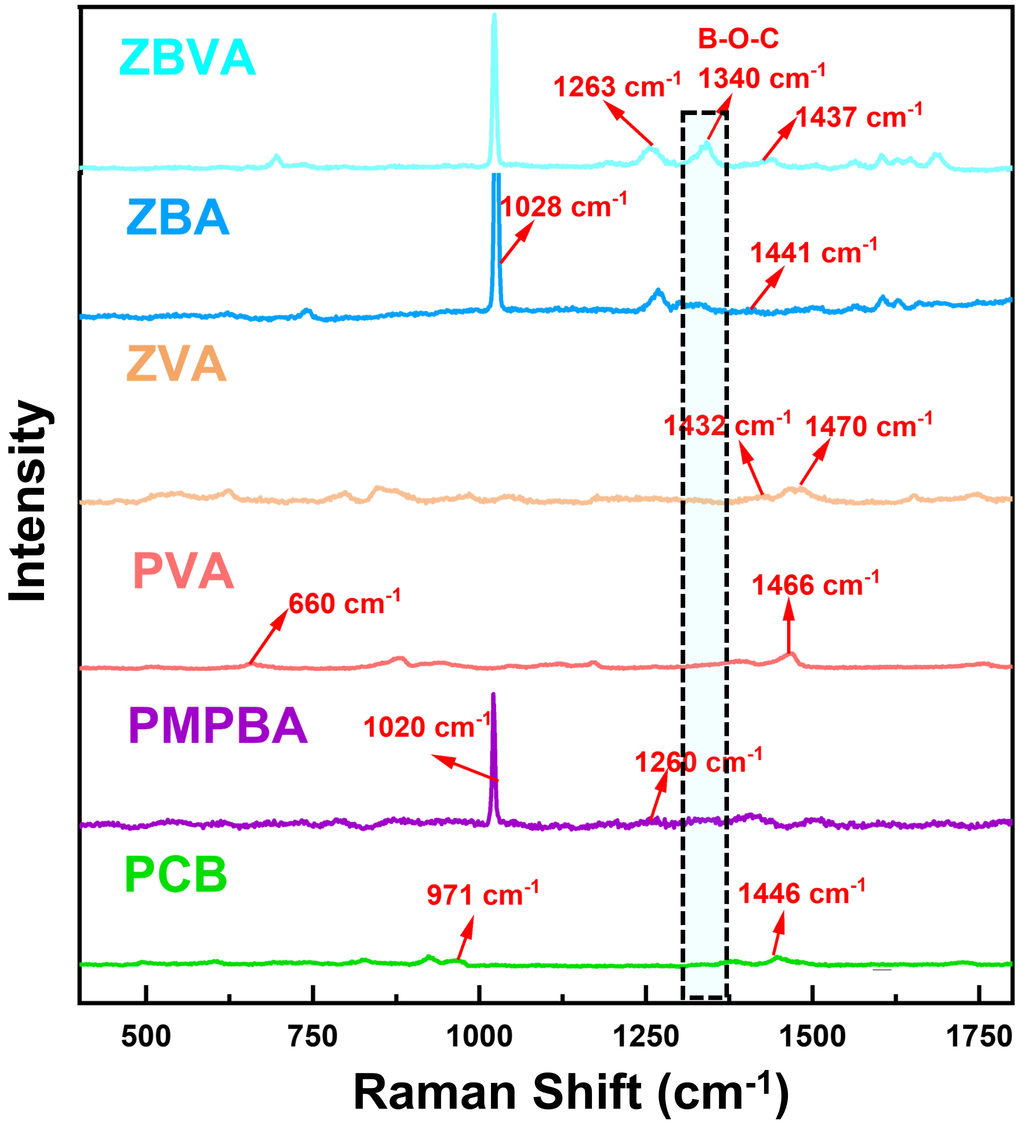

Supplementary Figure 5. The Raman test results and characteristic peaks of different hydrogels. Raman results showed that the spectrum of PCB hydrogel displayed peaks at 971 cm^{-1} and 1446 cm^{-1}, attributed to the antisymmetric stretching vibration of quaternary amine group (C_4N^+) and the stretching vibration of the carboxyl acid group (COO^-), respectively[8]. The spectrum of PMPBA hydrogel exhibited peaks at 1020 cm^{-1} (C=C in benzene ring breathing) and 1260 cm^{-1} (B-O stretching)[2,9,10]. The spectrum of PVA hydrogel displayed peaks at 660 cm^{-1} (O-H wagging) and 1466 cm^{-1} (C-OH bending)[5,11]. The characteristic peaks in the Raman spectra of ZVA, ZBA, ZBVA hydrogels demonstrated their successful preparation. The spectrum of ZVA hydrogel presented peaks at 1432 cm^{-1} (COO- stretching) and 1470 cm^{-1} (C-OH bending), while the spectrum of ZBA hydrogel exhibited peaks at 1028 cm^{-1} (C=C in benzene ring breathing) and 1441 cm^{-1} (COO^- stretching). Finally, the presence of alcoholic hydroxyl and phenoxyboronic acid groups in ZBVA hydrogel was demonstrated, owing to the formation of boronic ester bonds (B-O-C, characteristic peak at 1340 cm^{-1})[10,12].

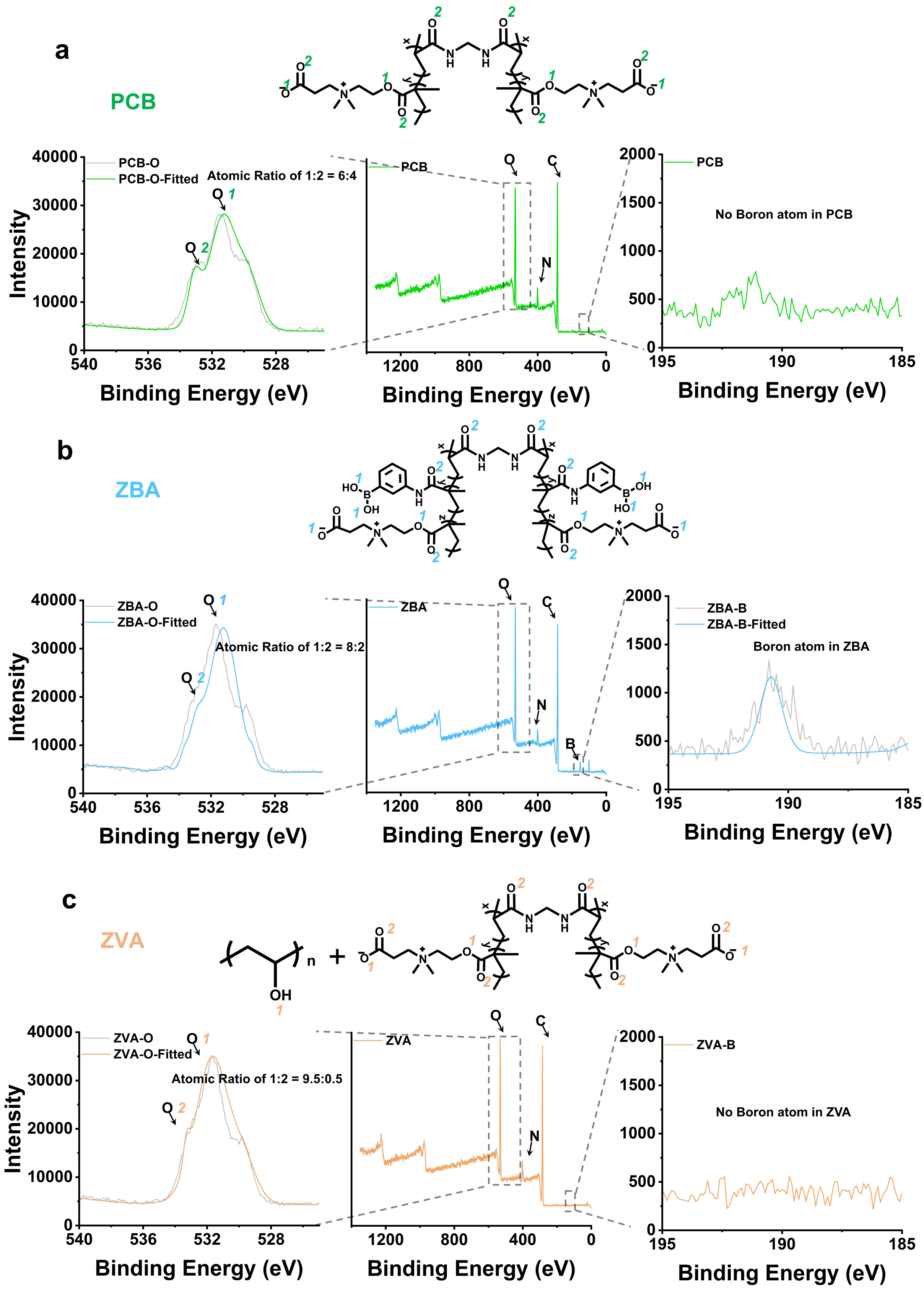

Supplementary Figure 6. The XPS tests and characteristic peaks of different hydrogels. XPS results show a typical peak from the phenylboric acid group in the ZBA hydrogel (**b**), while its absence in the PCB (**a**) and ZVA hydrogels (**c**). The ratio of typical peaks of the C-O_1 and C=O_2 bonds in the ZVA hydrogel (9.5:0.5) is increased compared

to that in the PCB (6:4), attributable to the additional C-O bonds in the PVA polymers into the ZVA. Moreover, the typical chemical compositions of C=O bonds in zwitterionic unit, B-O bonds in phenylboronic unit, and C-O bonds in the PVA, demonstrate the successful synthesis of ZBA and ZVA hydrogels.

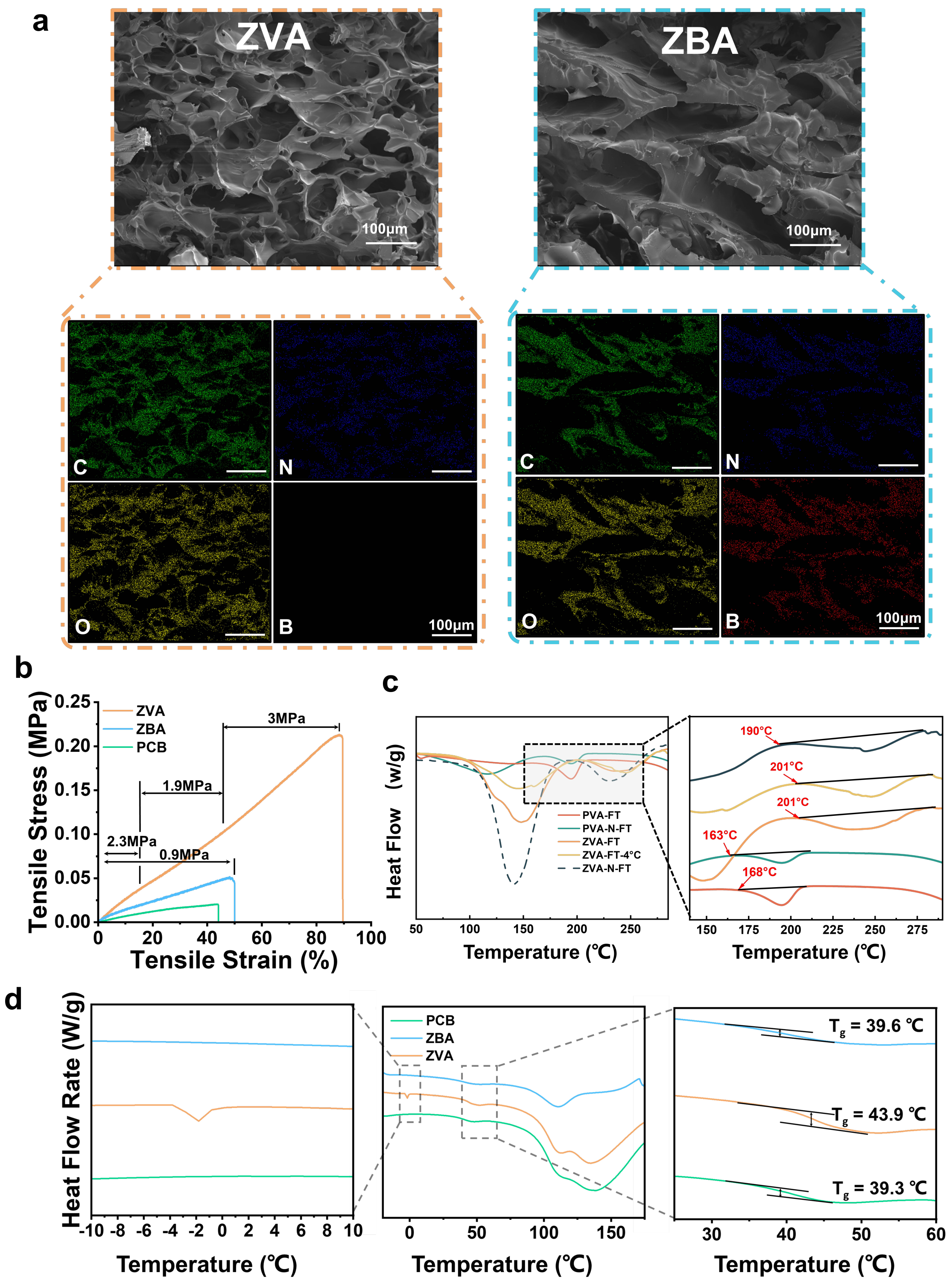

Supplementary Figure 7. SEM, Tensile Strength, and DSC tests of different hydrogels. a SEM images and SEM mapping images of bulk ZVA and ZBA hydrogels. Notably, the result shows the uniform distribution of C, N, and O elements in ZVA hydrogel (CBMA and PVA), as well as C, N, O, and B elements in ZBA hydrogel (CBMA and MPBA), indicating that the hydrogels are well synthesized without phase separation **b** Tensile strength test of bulk ZBA, ZVA hydrogels and control bulk PCB

hydrogel. ZBA and ZVA hydrogels exhibit both superior extensibility and tensile strength compared to pristine zwitterionic PCB hydrogel, owing to the phenylboric acid groups and PVA network, respectively. Notably, a constant modulus of ZBA hydrogel present its single chemical crosslinking network[13], while a continuously changing modulus of ZVA hydrogel reveal its IPN structure, because the crystalline domain and hydrogen bonds in PVA network served as sacrificial sites to dissipate energy during stretching[14]. **c,** DSC test of different hydrogels. To verify the physical crosslinking network by PVA, we supplemented the DSC tests and demonstrated the stable structure of ZVA hydrogel at 4°C (CTC preservation temperature). We constructed a series of samples for comparison as shown in **Supplementary Table 1**.

Supplementary Table 1. The detailed information of the tested samples

Name	Material and Process	Form	Photograph
PVA-N-FT	PVA polymer solutions without freeze-thaw cycles<br>(Polymers)	Solution	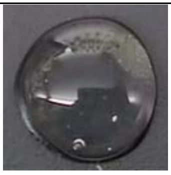
PVA-FT	PVA polymer solutions were repeated freeze-thaw cycles<br>(One physical crosslinking network)	Hydrogel	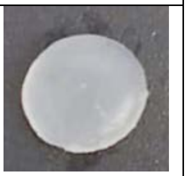
ZVA-N-FT	PVA polymer solutions were permeated into PCB network without freeze-thaw cycles (One chemical crosslinking network+ polymers)	Hydrogel	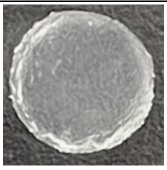
ZVA-FT	PVA polymer solutions were permeated into PCB network and repeated freeze-thaw cycles (One chemical crosslinking network+ one physical crosslinking network)	Hydrogel	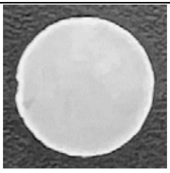
ZVA-FT-4°C	ZVA-FT hydrogels were incubated in PBS at 4°C for 7 days (One chemical crosslinking network+ one physical crosslinking network)	Hydrogel	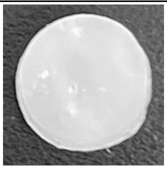

The melting peaks of all hydrogels were between 150-200°C for crystallinity

analysis. The crystallinity of PVA-FT (hydrogel) was significantly increased compared to PVA-N-FT (solution), according to 'methods' as below, indicating the domain-like crystalline manner as physical crosslinking sites for PVA hydrogel formation after the freeze-thaw process[15-17]. Moreover, it showed the melting point temperature: ZVA-FT > ZVA-N-FT > PVA-FT (single physical crosslinking network). The resulted suggested that there were two networks, including physical and chemical crosslinking networks, formed in the ZVA-FT hydrogel. After 7-day incubation in PBS, the melting point of ZVA-FT-4°C was negligible change compared with ZVA-FT, suggested that its stability at 4°C used in this preservation platform. Moreover, the fried-dry mass of ZVA hydrogel was stable *($\Delta M < 10$ mg)* during 7-day incubation at 4°C, indicating its 'IPN' structure.

Methods:

To calculate the crystallinity of PVA-based samples, the Supplementary equation (1) were used[16,17]:

$$f_c = \frac{\Delta H}{\Delta H°m} \times 100\% \qquad (1)$$

In the equation, f_c is degree of crystallinity, ΔH is heat fusion of PVA hydrogels, and $\Delta H°m$ is the thermodynamic enthalpy of melting of a 100% crystalline PVA which was reported as 150 J/g[18].

d DSC test of bulk ZBA, ZVA hydrogels and control bulk PCB hydrogel. The ZBA hydrogel exhibits similar curve and glass transition temperature (T_g) as well as the PCB hydrogel, whereas ZVA shows an additional endothermic peak at -0.8°C and a higher T_g, indicating its denser polymer chains owing to IPN structure, consistent with tensile strength results[19,20]

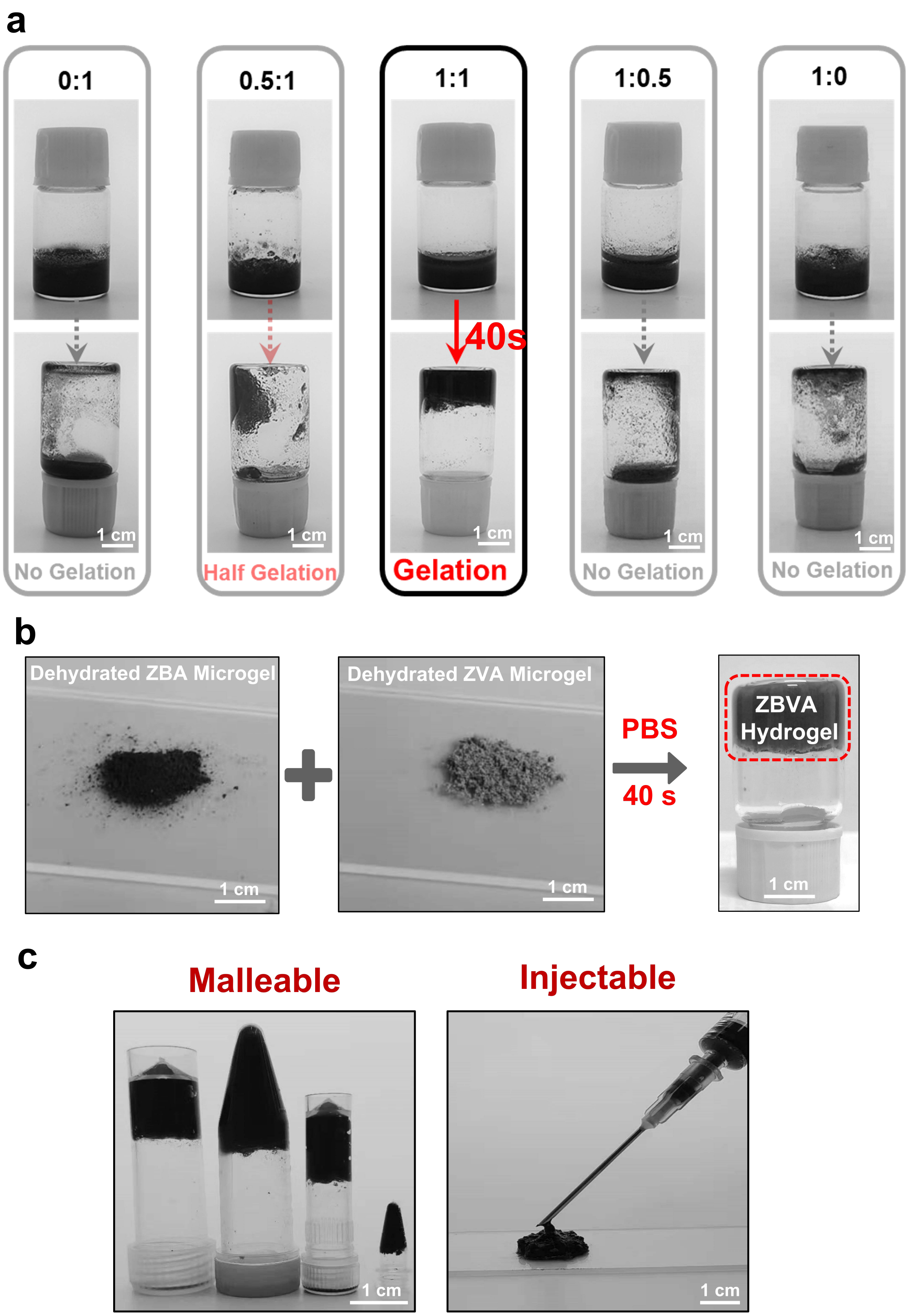

Supplementary Figure 8. Formation of ZBVA hydrogel. a The gelation behaviors of ZBVA hydrogels by different ratios of ZBA and ZVA microgel. **b** The rapid forming process of ZBVA hydrogel by two different hydrogels at 1:1 ratio in PBS solution. **c** The malleable and injectable ability of ZBVA hydrogel.

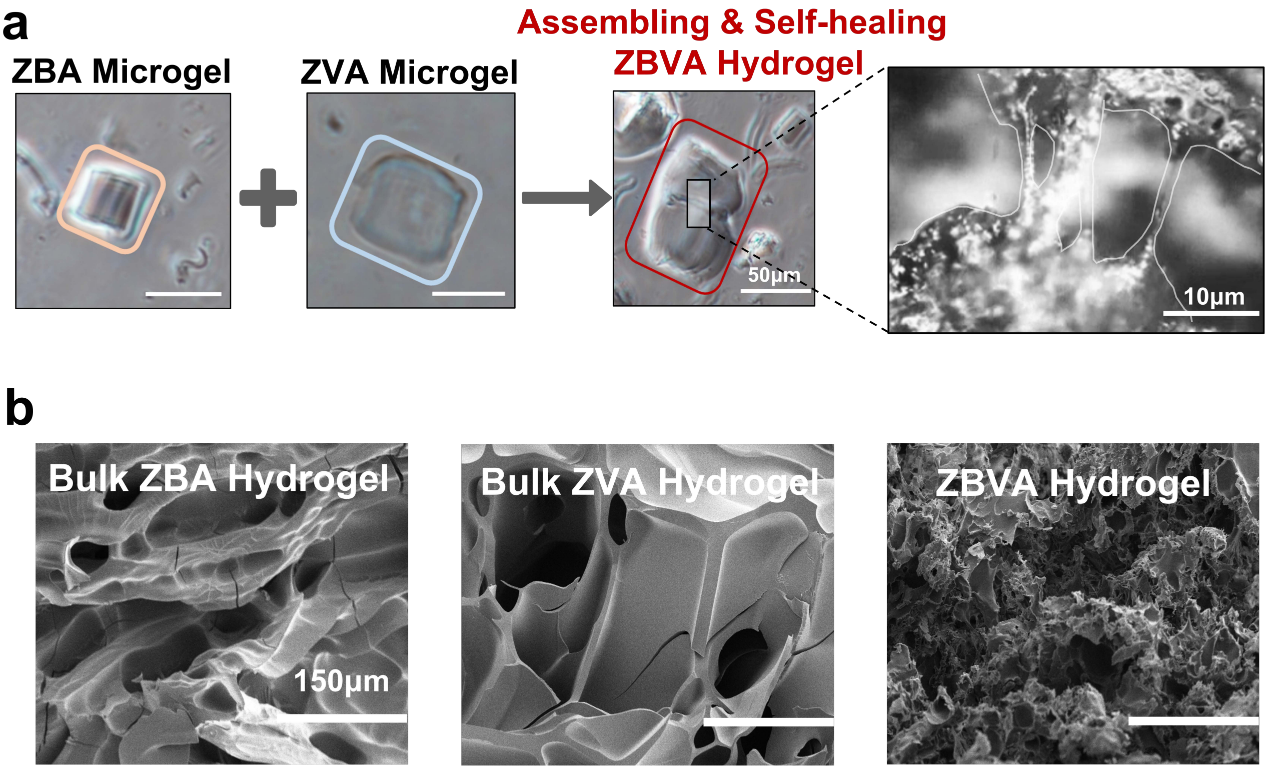

Supplementary Figure 9. The construction and microscopic graphics of ZBVA hydrogel. a The mild and rapid forming process of ZBVA hydrogel and microgel assembling process. **b** The SEM graphics of ZBA microgel, ZVA microgel, and ZBVA hydrogel.

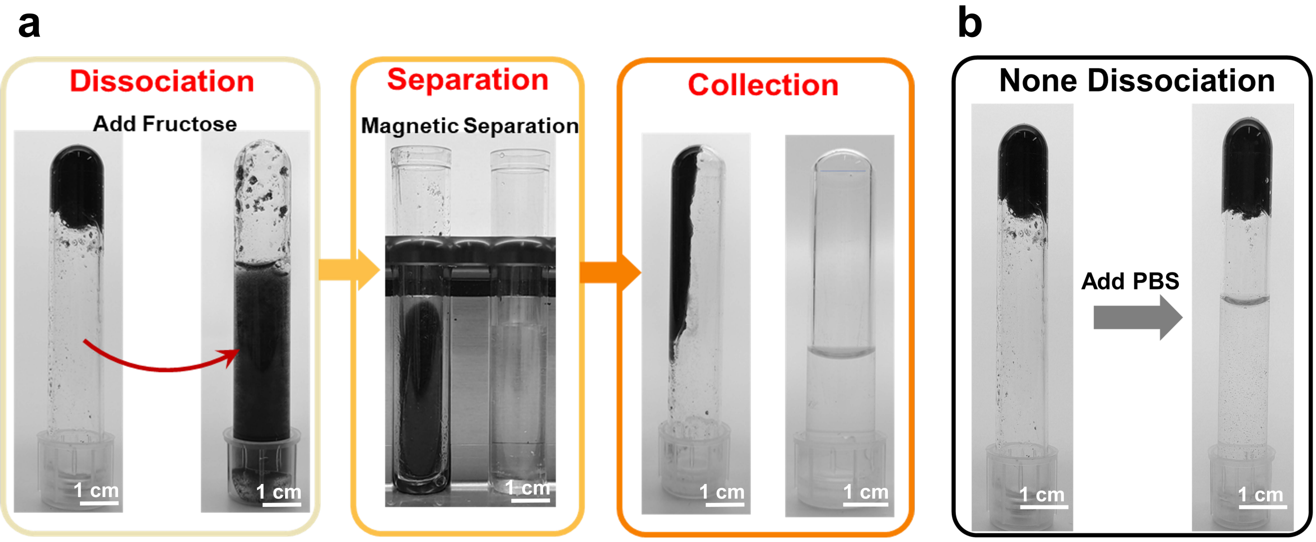

Supplementary Figure 10. The dissociation behaviors of ZBVA hydrogel. a The dissociation process of ZBVA hydrogel in fructose solution and its effortless collection by magnetic grate. **b** Stability of ZBVA hydrogel in PBS solution.

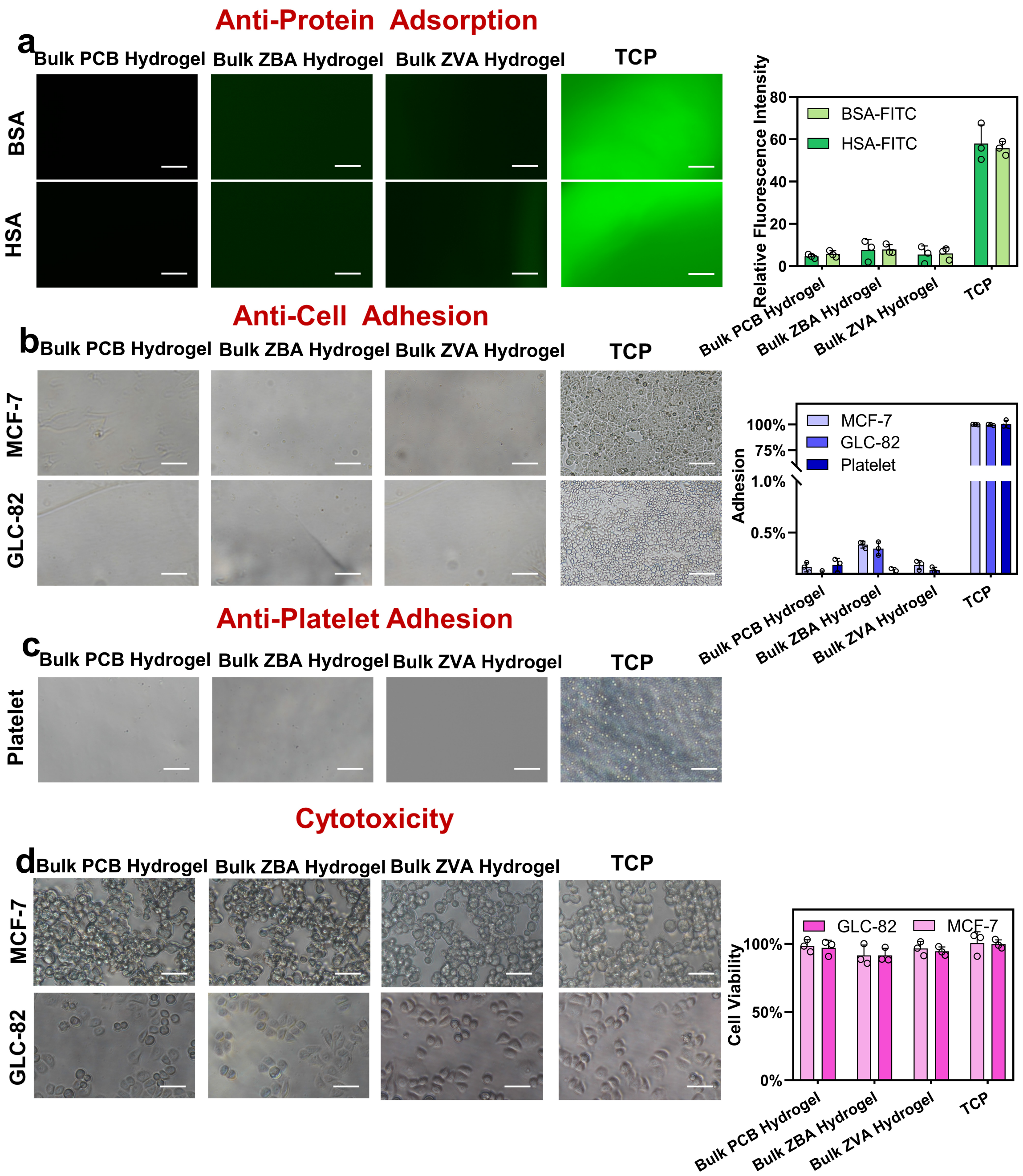

Supplementary Figure 11. The anti-biofouling properties and cytotoxicity of bulk ZBA and ZVA hydrogels compared with PCB hydrogel and TCP. a The protein (HAS and BSA) adsorption tests of ZBA and ZVA hydrogel. Scale bar is 100 μm. **b** The prevention of cell adhesion (MCF-7 and GLC-82 cells) and, **c,** Anti-platelet adhesion by ZBA and ZVA hydrogels. Scale bar is 100 μm. **d** Cell morphology and viability of MCF-7 and GLC-82 cells cultured in the extracts of different hydrogels compared with culture medium. Scale bar is 20 μm. Values represent mean ± s.d. of n=3 independent experiments.

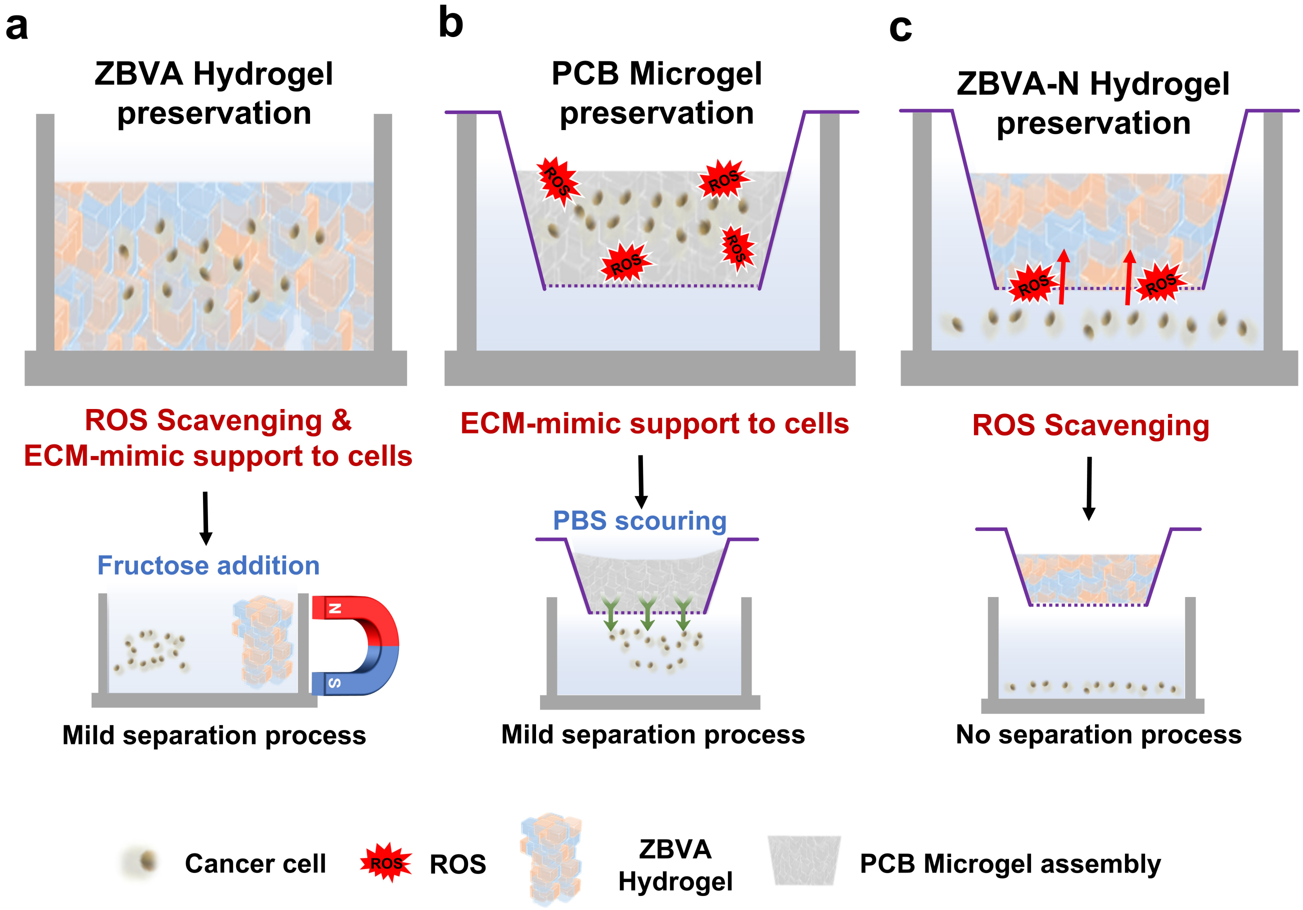

Supplementary Figure 12. The three preservation methods for analyzing the cell long-term preservation mechanism of ZBVA hydrogel. **a** Cell-embedded ZBVA hydrogel system for both ROS scavenging and ECM mimicking. **b** PCB microgel preservation system for 3D ECM-mimic support. **c** ZBVA-N hydrogel system, wherein cells are not encapsulated but out of the well for ROS scavenging.

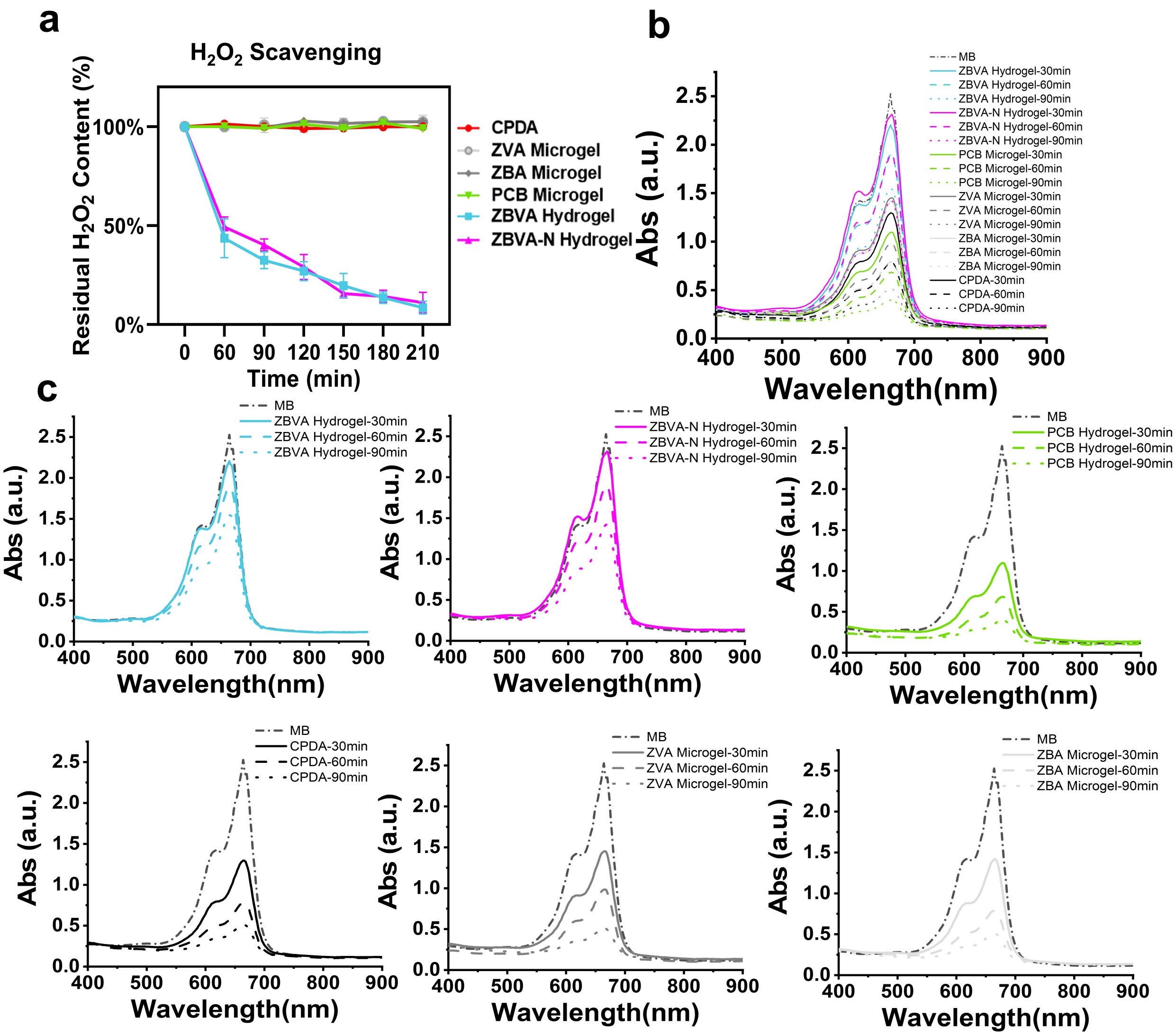

Supplementary Figure 13. a The H_2O_2 scavenging by different systems. **b, c** The absorption spectra changes of MB solution in different systems at 400-900 nm. For **a**, values represent mean ± s.d. of n=3 independent experiments.

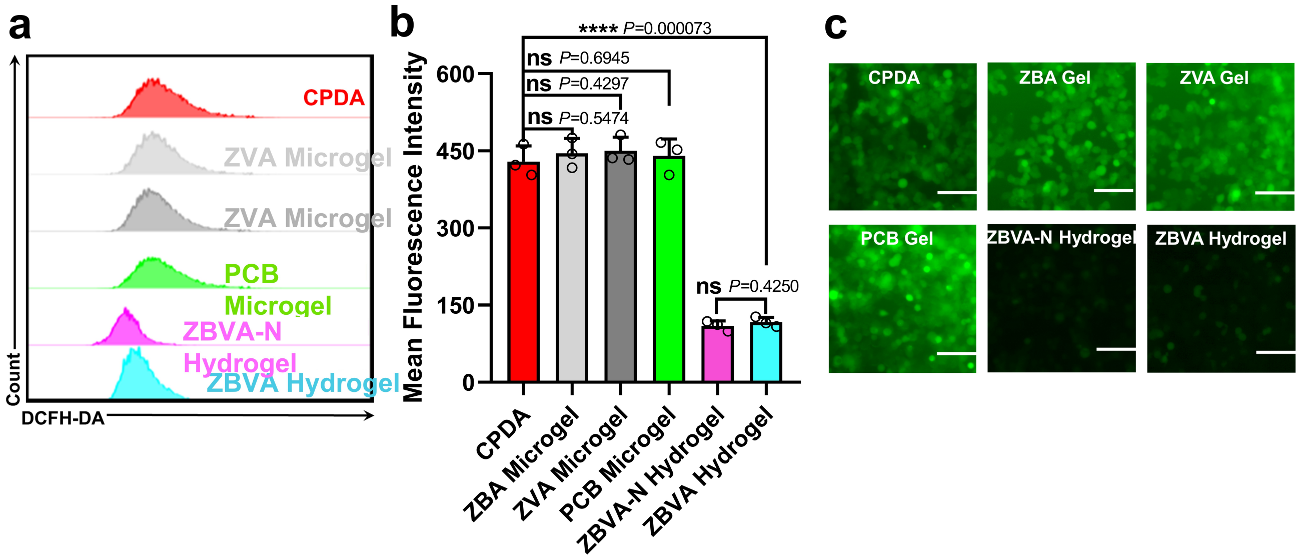

Supplementary Figure 14. The intracellular ROS scavenging detected by flow cytometry (a, b) and florescence microscope (c). The fluorescence signals are detected by DCFH-DA probe. Scale bar is 50 μm. Values represent mean ± s.d. of n=3 independent experiments and the two-tailed Student's *t*-test related *p* values are indicated.

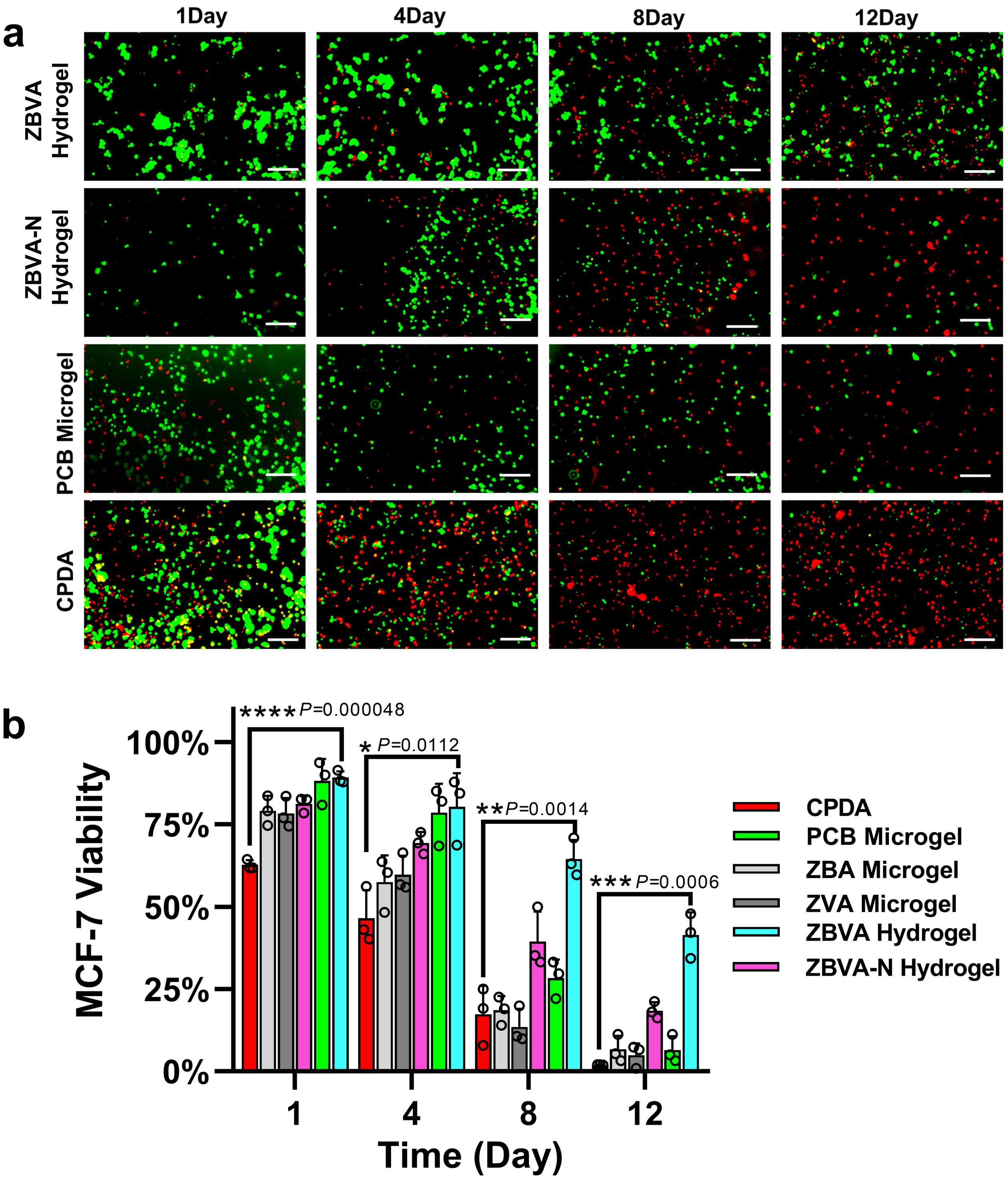

Supplementary Figure 15. a, b The fluorescence images (**a**) and viability (**b**)of cells preserved in different systems during 12 days using the live/dead staining assay. Scale bar is 50 μm. For **b**, values represent mean ± s.d. of n=3 independent experiments and the two-tailed Student's *t*-test related *p* values are indicated.

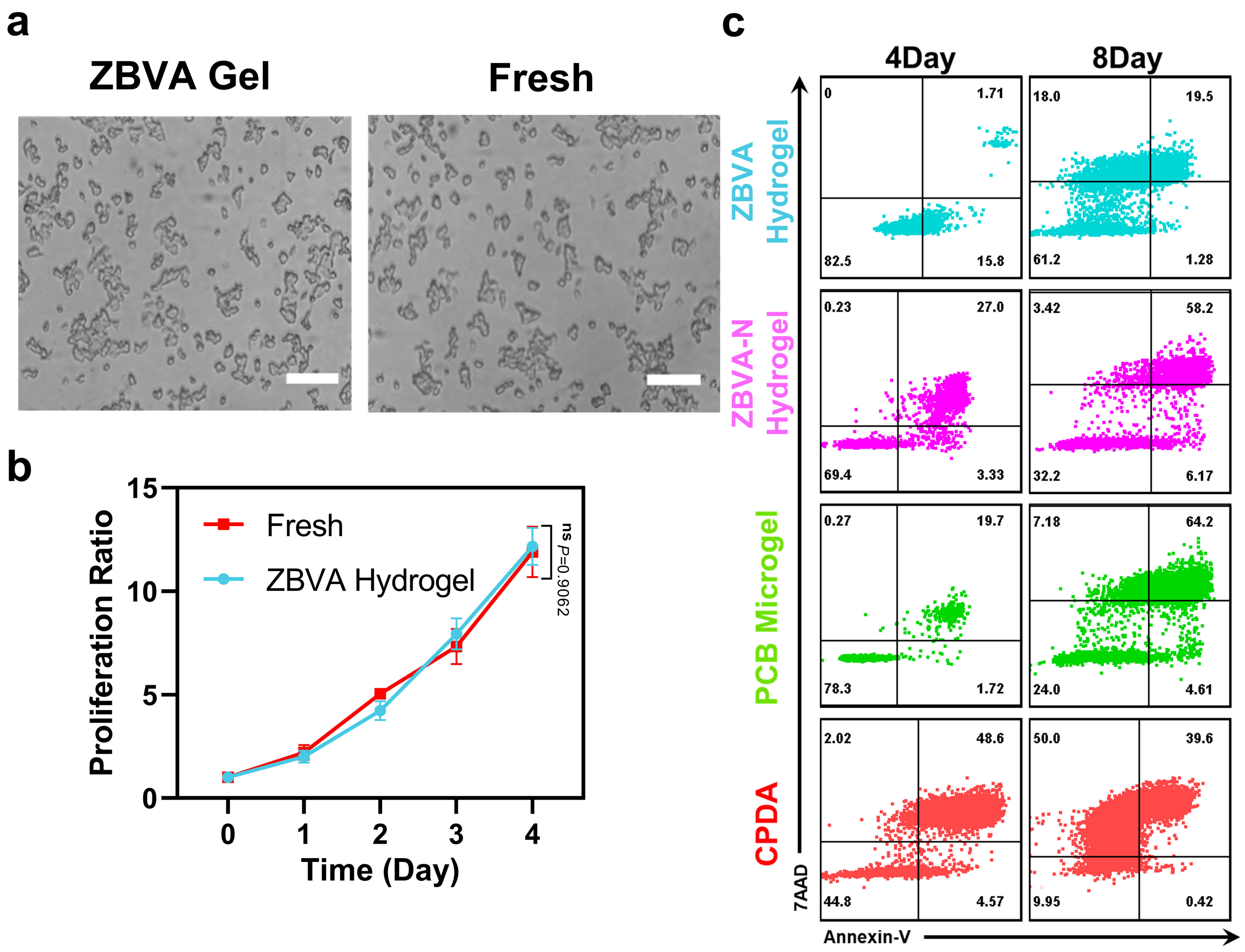

Supplementary Figure 16. a, b, The morphology (**a**) and proliferation rate (**b**) of recovered cells from ZBVA hydrogel, the scale bar is 50μm. **c** The apoptosis and viability of cells is reconfirmed by flow cytometry. For **b**, values represent mean ± s.d. of n=3 independent experiments and the two-tailed Student's *t*-test related *p* values are indicated.

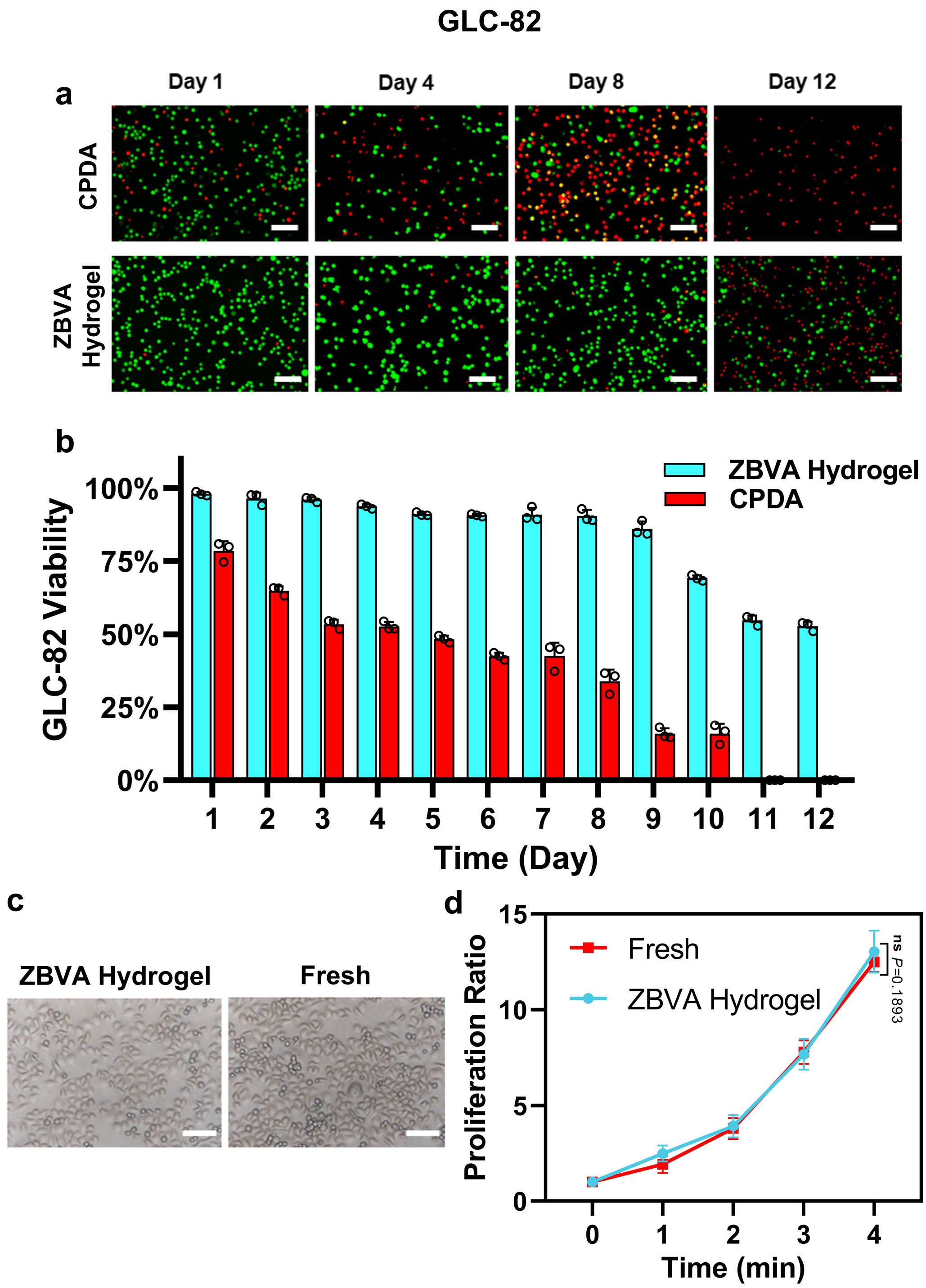

Supplementary Figure 17. The viability of GLC-82 cells preserved in ZBVA hydrogel and CPDA solution a The fluorescence microscope images of cell live/dead staining during 12days storage, the scale bar is 50μm. **b** The viability of cells calculated from live/dead staining graphs. **c, d** The functionalities like morphology (**c**), and proliferation rate (**d**) of recovered GLC-82 cells, the scale bar is 50μm. For **d**, values represent mean ± s.d. of n=3 independent experiments and the two-tailed Student's *t*-test related *p* values are indicated.

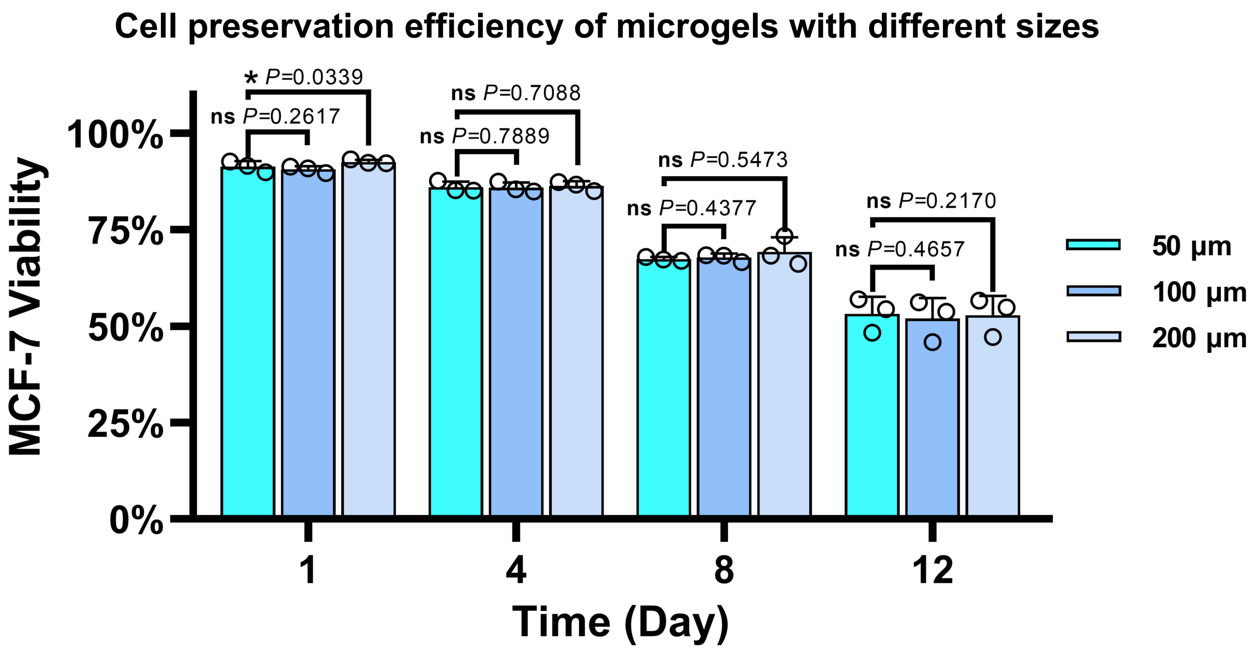

Supplementary Figure 18. Cell preservation efficiency of ZBVA hydrogel with different sizes of microgels. Values represent mean ± s.d. of n=3 independent experiments and the two-tailed Student's t-test related p values are indicated.

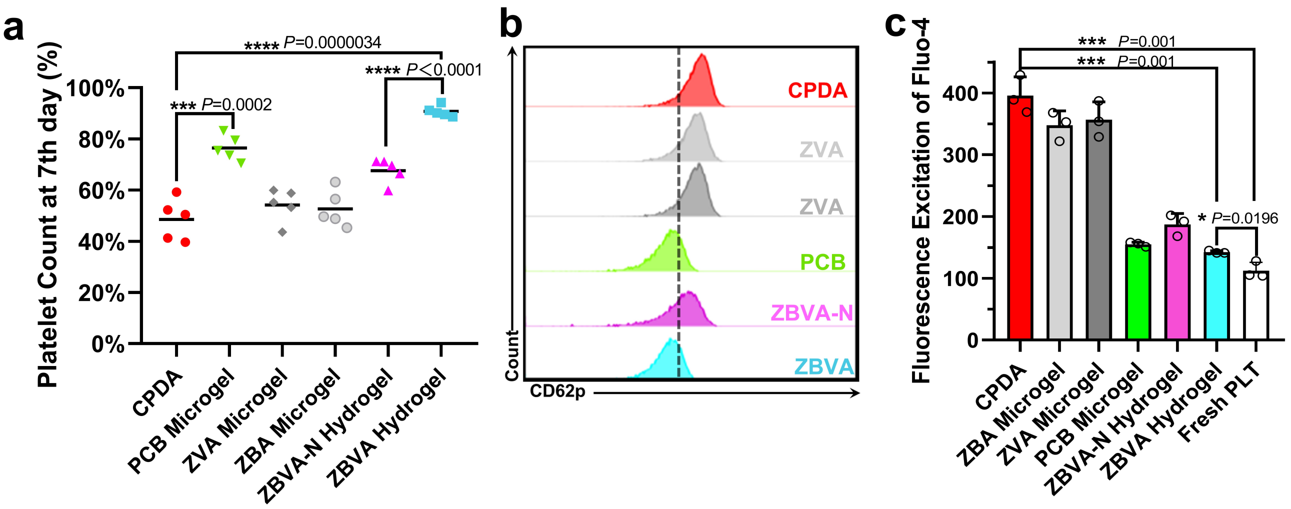

Supplementary Figure 19. a The changing process of platelet preserved in CPDA solution or ZBVA hydrogel during 7-day storage. **b** The platelet activation level on the 7th day are assessed by percentage of P-selectin by flow cytometry. **c** The changes of platelet cytosolic free calcium in different groups on the 7th day assessed by the fluorescence intensity of Fluo-4 probe. For **a** and **c**, values represent mean ± s.d. of n=3 independent experiments and the two-tailed Student's *t*-test related *p* values are indicated.

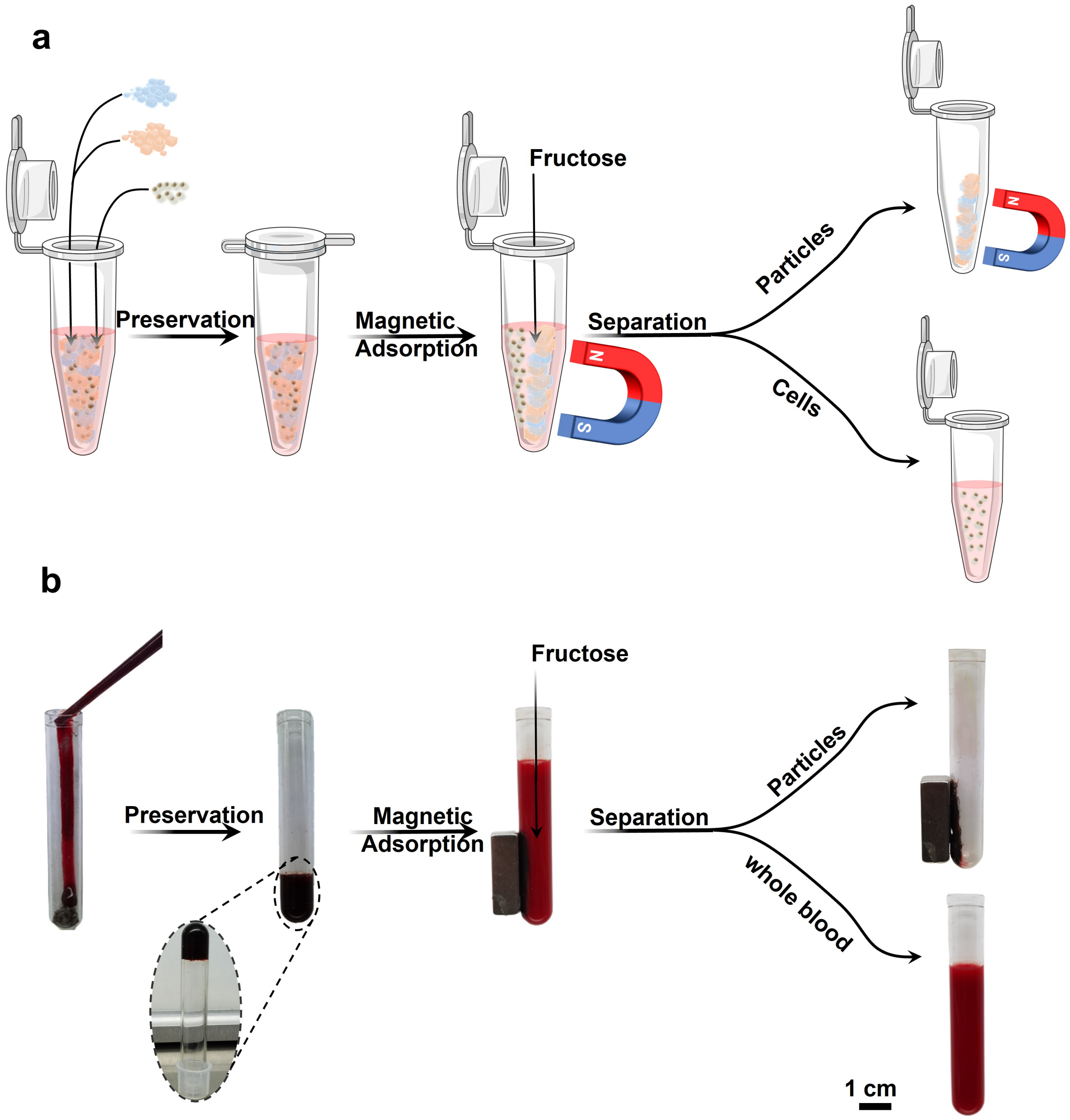

Supplementary Figure 20. The process of preserving cells in ZBVA hydrogel. a The illustration of preservation and recollection process of cells in ZBVA hydrogel. **b** The images of preserving whole blood specimen in ZBVA hydrogel.

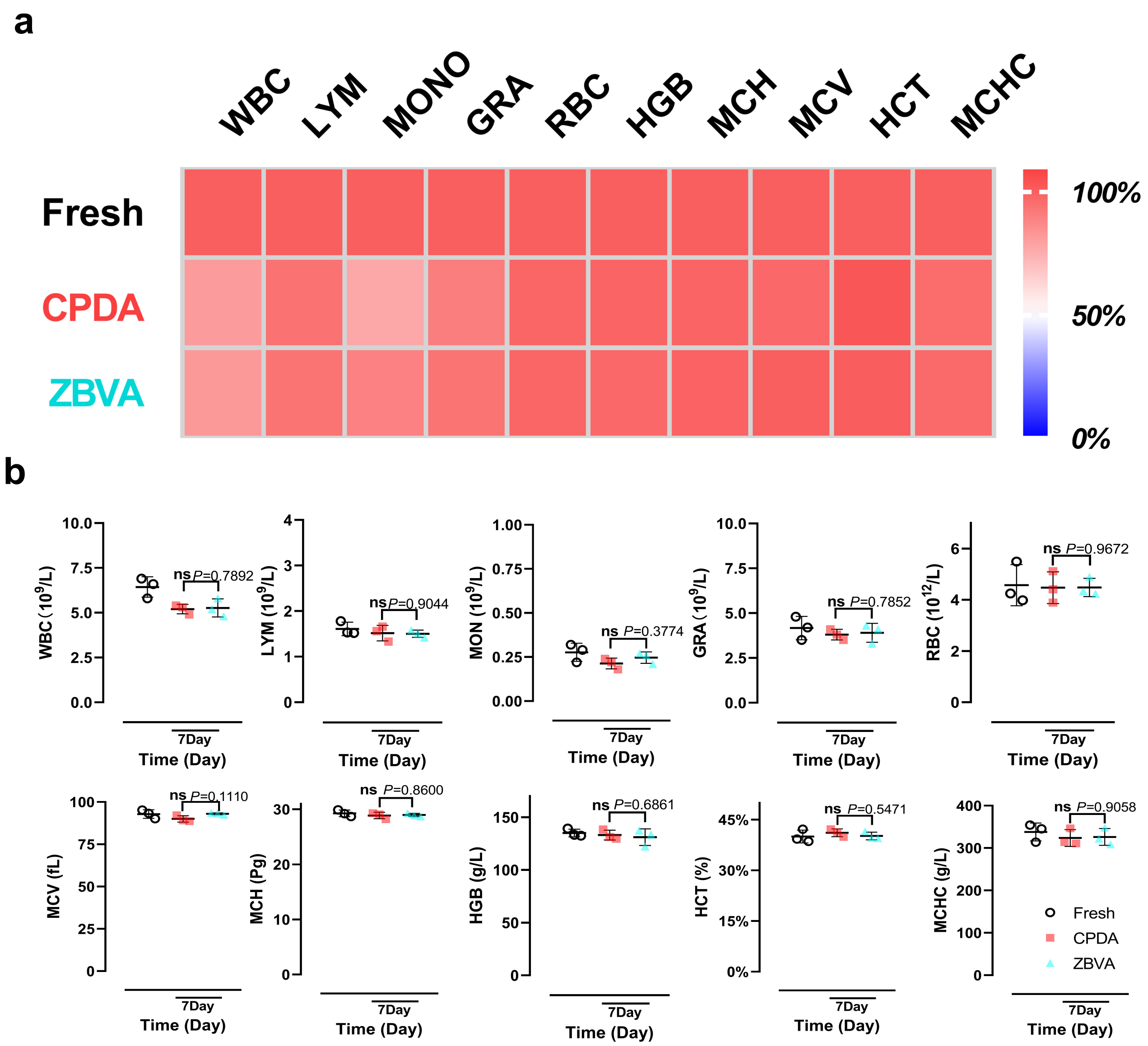

Supplementary Figure 21. The hematology analysis of whole blood specimen preserved in CPDA solution and ZBVA hydrogel. a,b The statistical heatmap (**a**) and histograms (**b**) of hematology analysis. Fresh and 7-day storage blood specimens are tested. WBC: white blood cell; LYM: lymphocyte; MONO: monocyte; GRA: granulocyte; RBC: red blood cell; HGB: hemoglobin; MCH: mean corpuscular hemoglobin; MCV: mean corpuscular volume; HCT: hematocrit; MCHC: mean corpuscular hemoglobin concentration. For **b**, values represent mean ± s.d. of n=3 independent experiments and the two-tailed Student's *t*-test related *p* values are indicated.

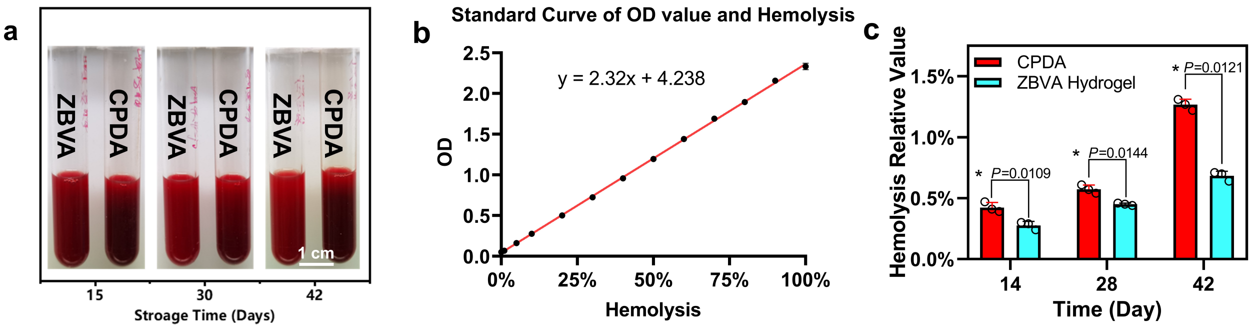

Supplementary Figure 22. a The images of preserved blood specimens during 42 days preservation. **b** The standard curve of OD value and RBCs hemolysis. **c** The histogram of hemolysis value of RBCs preservation. For **b** and **c**, values represent mean ± s.d. of n=3 independent experiments and the two-tailed Student's *t*-test related *p* values are indicated.

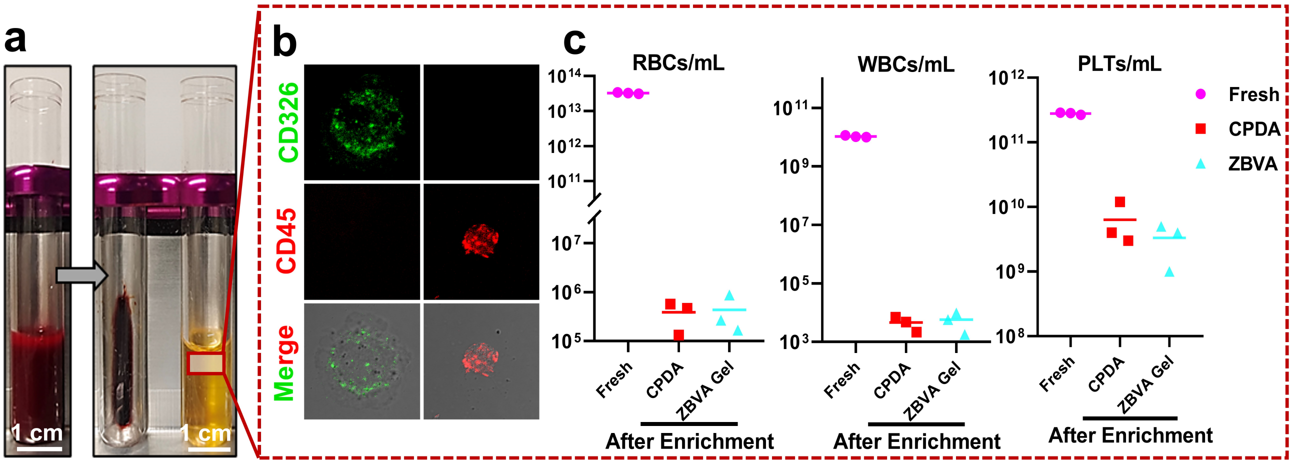

Supplementary Figure 23. a The separation of model CTCs from whole blood specimen by using CTC enrichment kit (CTCs EasySep kit from StemCell). **b, c** The confocal fluorescence images of model CTCs ($CD326^{+}$, $CD45^{-}$) and WBCs ($CD326^{-}$, $CD45^{+}$) and the number of RBCs, WBCs, and PLTs in the enriched solution (**c**). For **c**, values represent mean ± s.d. of n=3 independent experiments and the two-tailed Student's *t*-test related *p* values are indicated.

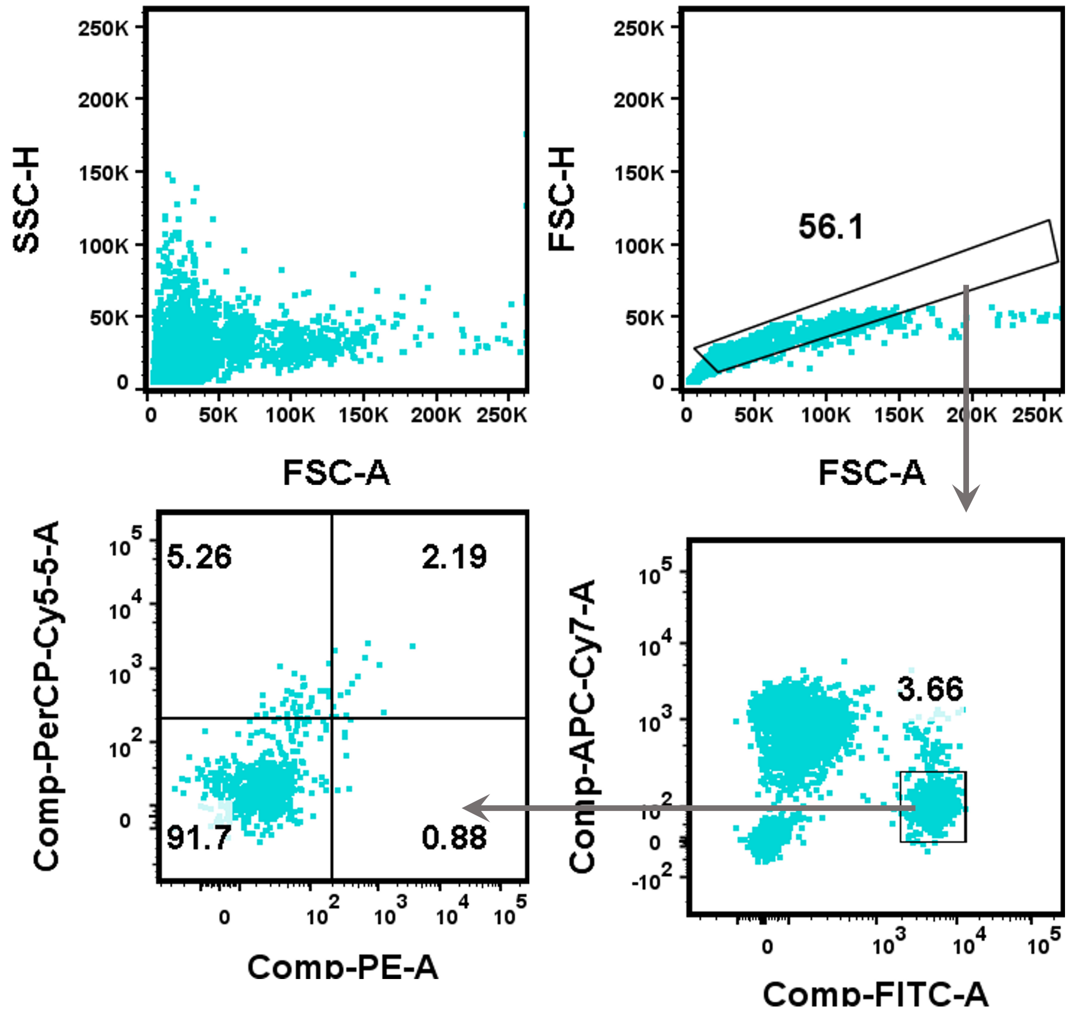

Supplementary Figure 24. Gating method of cell apoptosis and live/dead analysis. Representative FACS profiles are shown for analyzing apoptosis and live/dead model CTCs. Consecutive gating was applied.

Viability of CTCs in fresh patient blood

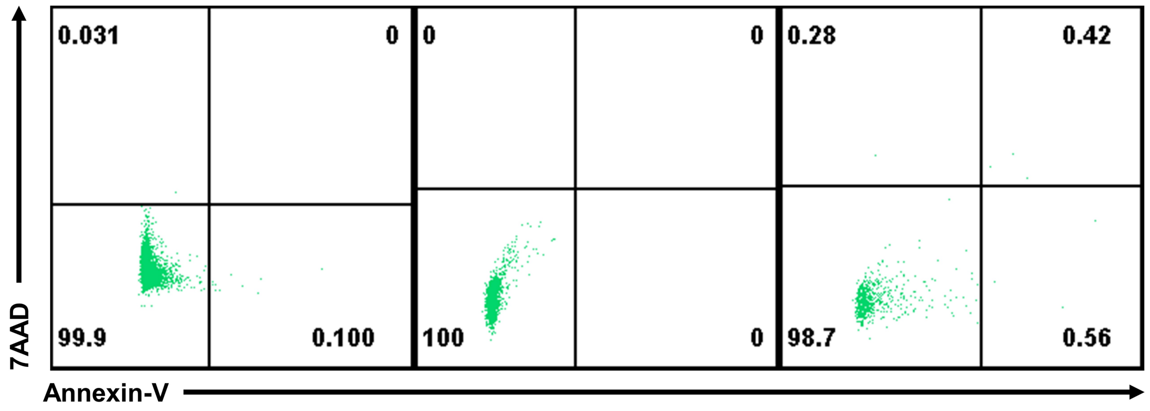

Supplementary Figure 25. The viability of model CTCs in fresh patient blood.

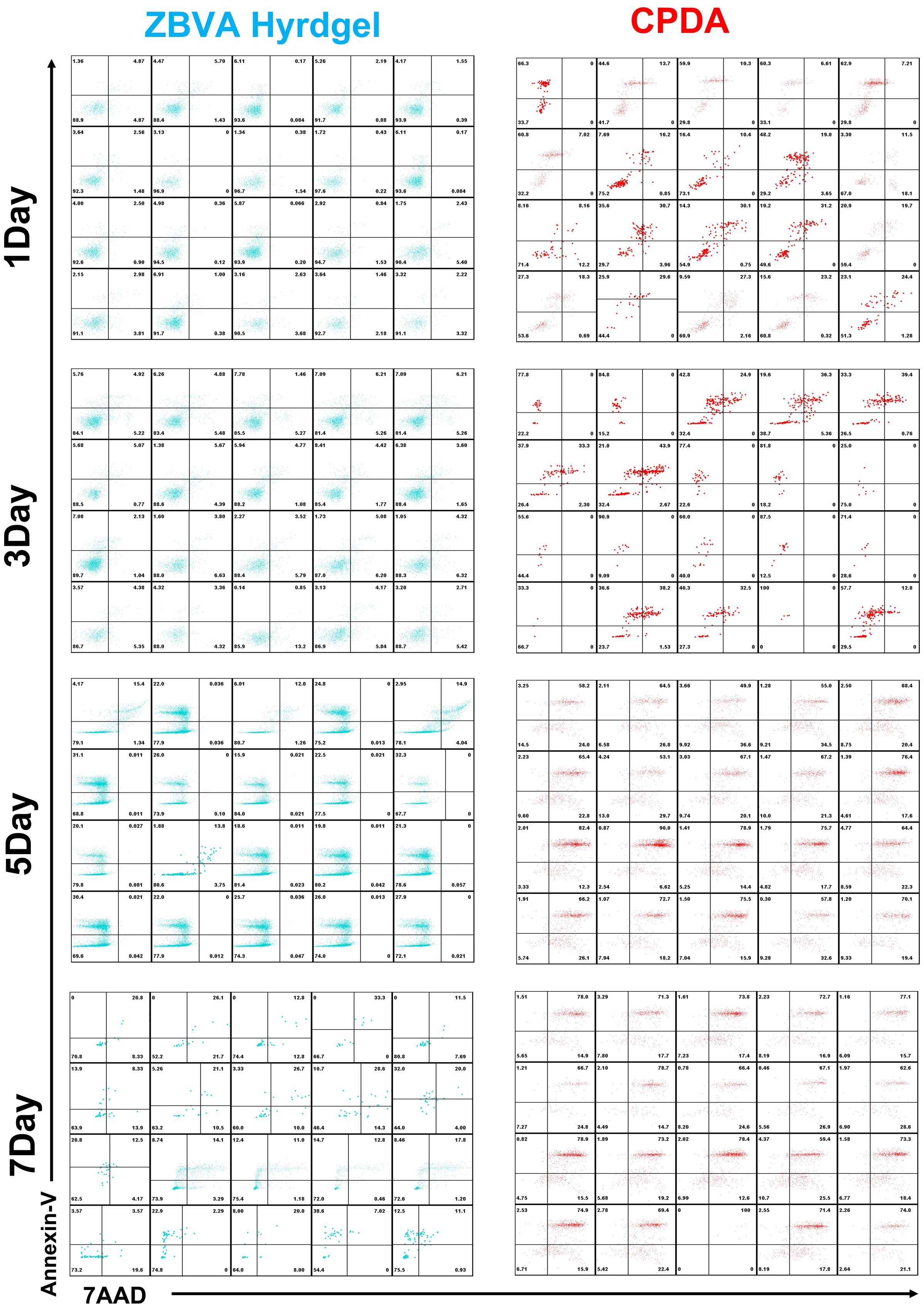

Supplementary Figure 26. Viability of model CTCs preserved in ZBVA hydrogel system and CPDA solution. FACS profiles for testing apoptosis and live/dead of the enriched model CTCs from whole blood specimens of 20 different breast cancer patients during 7 days.

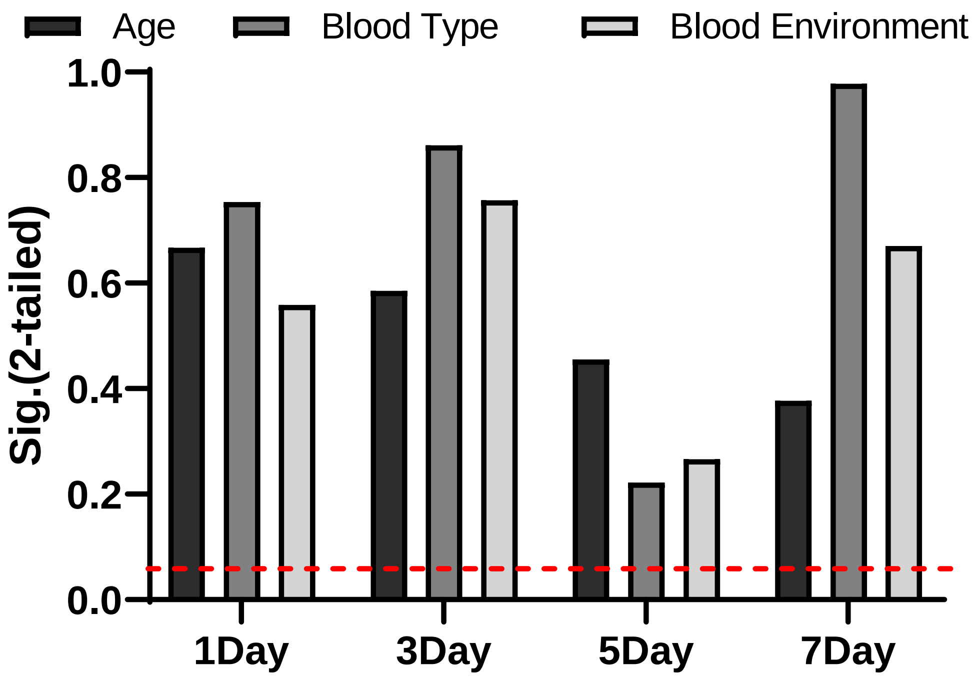

Supplementary Figure 27. The correlation between Age, Blood Type, Blood Environment (from various stage cancer patients) of patients and corresponding model CTC viability (analyzed by SPSS software) during 7 days preservation.

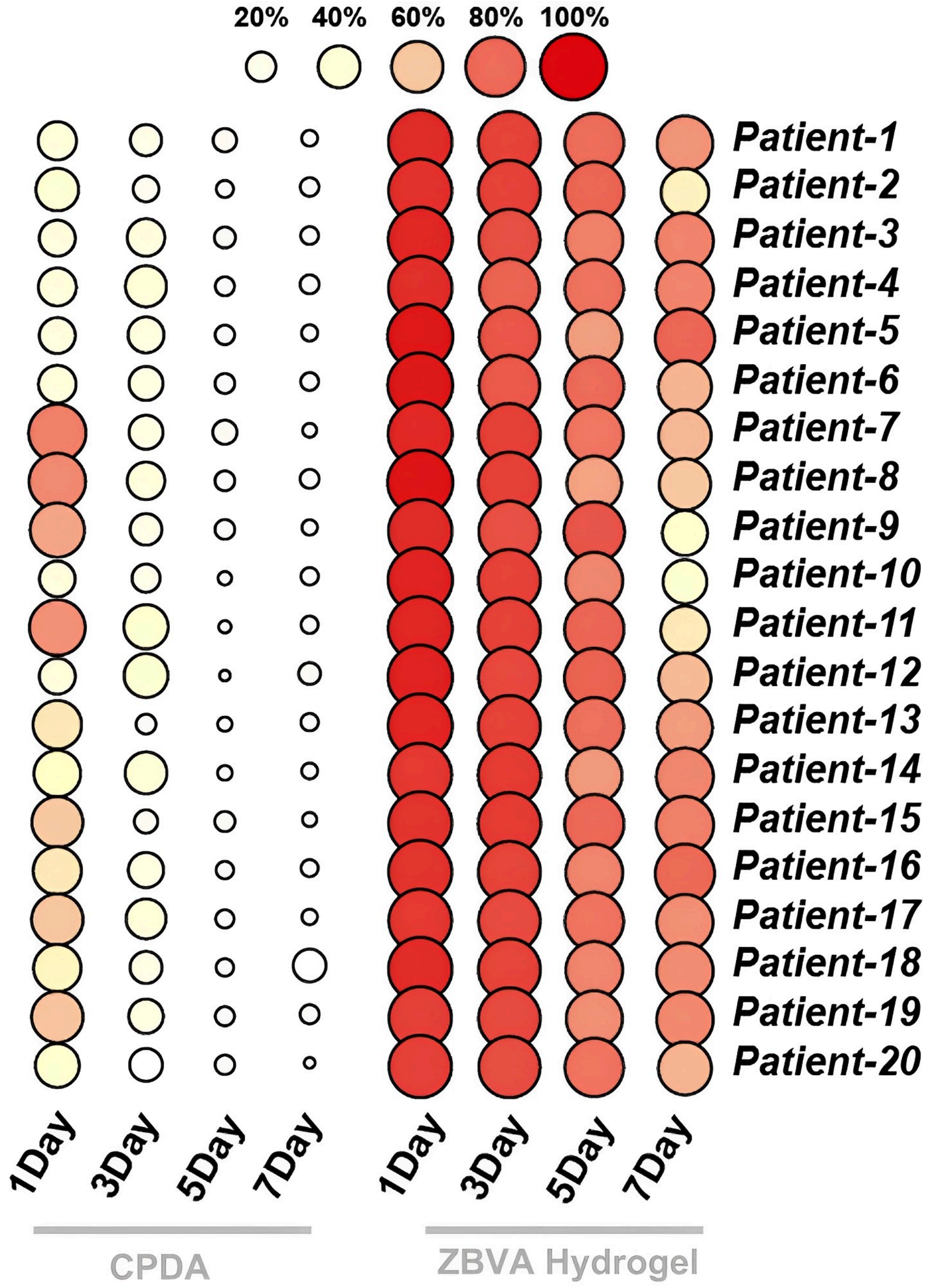

Supplementary Figure 28. The heatmap of model CTC viability during 7 Days storage of 20 patients by preserved in CPDA solution and ZBVA hydrogel.

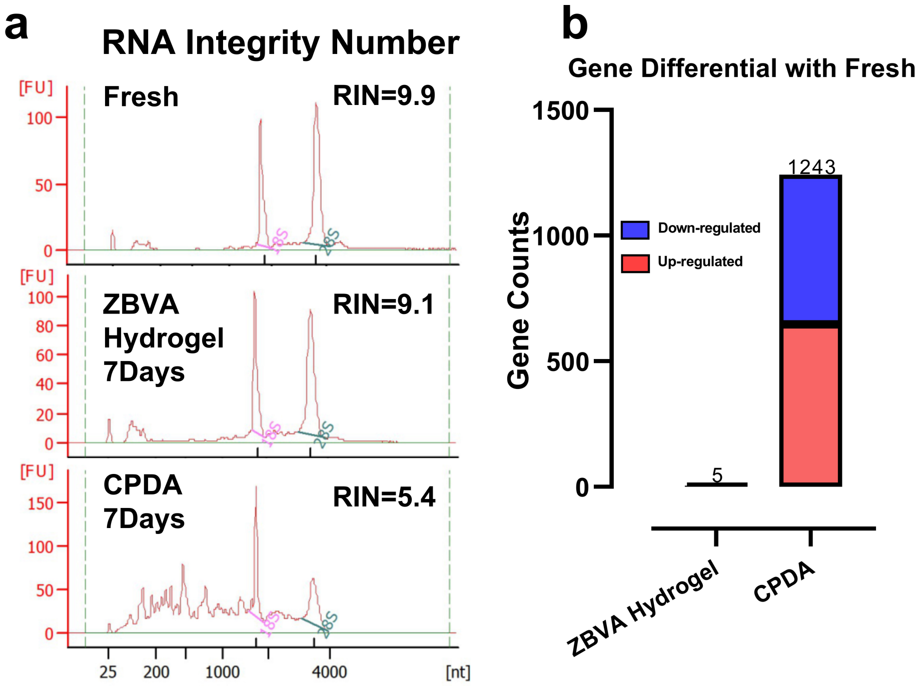

c

TOP 30 Changed genes	
UP	Down
RPL41	*AHNAK*
RPLP2	*HMCN1*
RPL13A	*DSP*
RPS19	*PRKDC*
RPL36	*THBS1*
RPL37	*FLNB*
RPS21	*COL12A1*
RPS6	*TRRAP*
RPS27	*TMEM64*
RPS28	*DYNC1H1*
RPL13	*ADCY1*
RPL35A	*UBR4*
RPS16	*CERS2*
RPL39P3	*IGF2R*
RPL12	*NOTCH2*
RPL32	*KMT2D*
RPL24	*GTF2I*
RPL23A	*CDC42BPB*
RPL35	*ATP1A1*
RPL14	*EPPK1*
RPL39	*NFAT5*
RPL11	*ABHD2*
RPL27A	*HSPG2*
RPS27A	*TRIO*
RPS15A	*NOTCH3*
RPS8	*UBE4A*
COX7C	*GSN*
UBA52	*XRN2*
RPL34	*MCM2*
MT2A	*SOGA1*

Supplementary Figure 29. RNA sequencing analysis of model CTCs isolated from whole blood specimens. a The representative data shows the distribution traces of total RNA of model CTCs in different preservation system. **b** The significant changing of gene expression of model CTCs in different preservation system. **c** The top 30 changed genes of model CTCs preserved in CPDA solution.

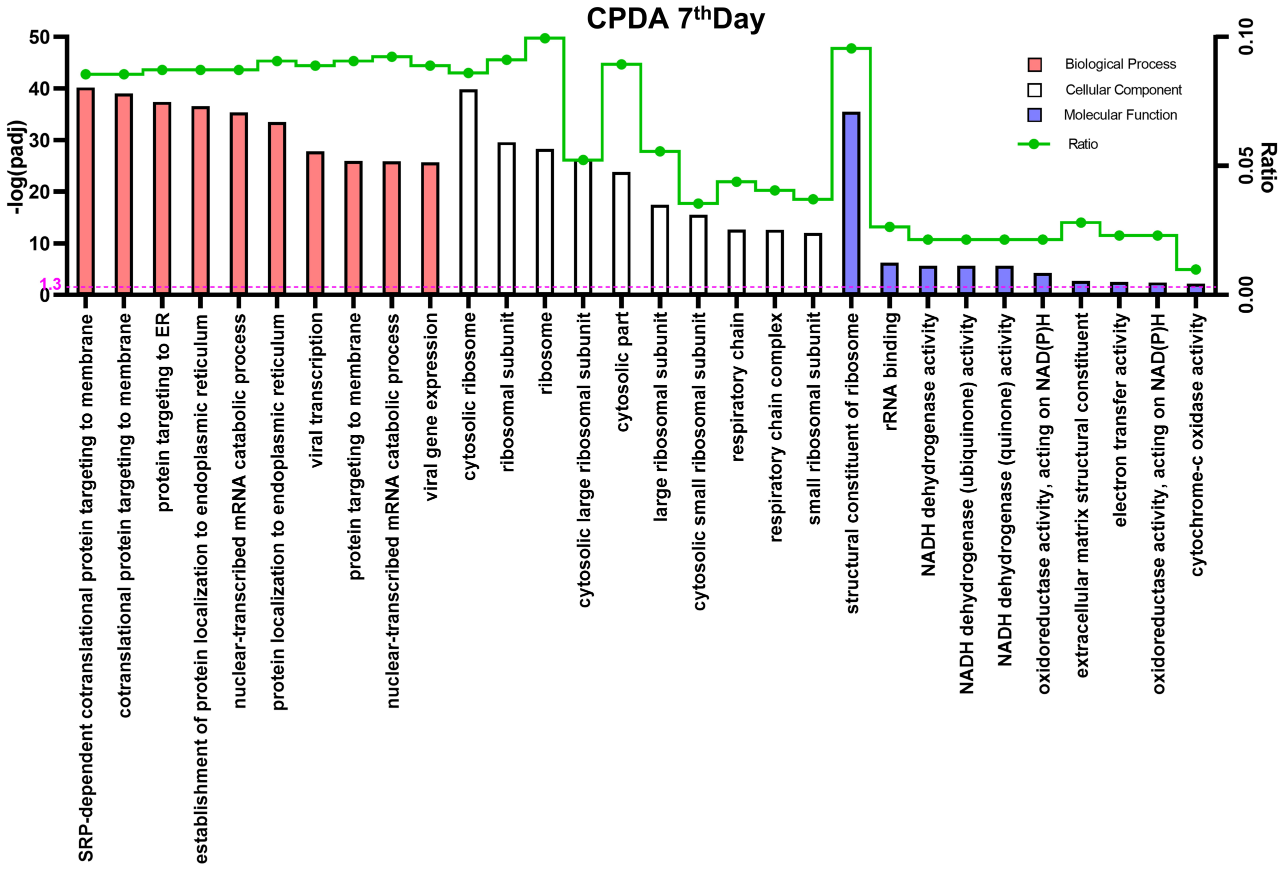

Supplementary Figure 30. The GO enrichment of model CTCs preserved in CPDA solution. Top 10 significant changes of biological processes (BP), cellular component (CC), and molecular function (MF) in model CTCs preserved in CPDA solution at 7th day. The y axis on the left indicates the significance score shown as -log(padj). The right y axis indicates the ratio of the number of GO pathway correlated genes that map to the total significant expressed genes (green line). The pink dashed line indicates where the -log(padj) equals 1.3, which is the boundary score between significance and non-significance.

Supplementary Table 2. The detailed information of Antibodies

Name	Manufacturer	Clone	Cat.number
CD45 Monoclonal Antibody APC	Biolegend	2D1	368516
CD62P (P-Selectin) Monoclonal Antibody PE	Invitrogen	Psel.KO2.3	12-0626-82
CD326 (EpCAM) Monoclonal Antibody Alexa Fluor 488	Invitrogen	MH99	53-8326-42

Supplementary References

1. Zhang, Z., *et al.* Nonfouling Behavior of Polycarboxybetaine-Grafted Surfaces: Structural and Environmental Effects. *Biomacromolecules* **9**, 2686-2692 (2008).
2. Piergies, N., Proniewicz, E., Ozaki, Y., Kim, Y. & Proniewicz, L.M. Influence of substituent type and position on the adsorption mechanism of phenylboronic acids: infrared, Raman, and surface-enhanced Raman spectroscopy studies. *J. Phys. Chem. A* **117**, 5693-5705 (2013).
3. Das, R.K. & Mohapatra, S. Highly luminescent, heteroatom-doped carbon quantum dots for ultrasensitive sensing of glucosamine and targeted imaging of liver cancer cells. *J Mater Chem B* **5**, 2190-2197 (2017).
4. Mondal, S. & Banthia, A.K. Low-temperature synthetic route for boron carbide. *J. Eur. Ceram. Soc.* **25**, 287-291 (2005).
5. Prosanov, I.Y. & Matvienko, A.A. Study of PVA thermal destruction by means of IR and Raman spectroscopy. *Physics of the Solid State* **52**, 2203-2206 (2010).
6. Anandalli, M.H., *et al.* Mechanical and thermal studies of brilliant green dye doped poly(vinyl alcohol) polymer composite. in *Dae Solid State Physics Symposium 2018* (2019).
7. Spitler, E.L. & Dichtel, W.R. Lewis acid-catalysed formation of two-dimensional phthalocyanine covalent organic frameworks. *Nat. Chem.* **2**, 672-677 (2010).
8. Sun, F., *et al.* Stealth surface modification of surface-enhanced Raman scattering substrates for sensitive and accurate detection in protein solutions. *ACS Nano* **9**, 2668-2676 (2015).
9. Li, S., Zhou, Q., Chu, W., Zhao, W. & Zheng, J. Surface-enhanced Raman scattering behaviour of 4-mercaptophenyl boronic acid on assembled silver nanoparticles. *Phys. Chem. Chem. Phys.* **17**, 17638-17645 (2015).
10. Hou, X., Li, Z. & Zhang, Z. Selectively Producing Acetic Acid via Boric Acid-Catalyzed Fast Pyrolysis of Woody Biomass. *Catalysts* **11**(2021).
11. Cooney, T.F., Wang, L., Sharma, S.K., Gauldie, R.W. & Montana, A.J. Raman spectral study of solid and dissolved poly(vinyl alcohol) and ethylene-vinyl

alcohol copolymer. *J. Polym. Sci., Part B: Polym. Phys.* **32**, 1163-1174 (1994).

12. Stagno, V., *et al.* Non-Invasive Assessment of PVA-Borax Hydrogel Effectiveness in Removing Metal Corrosion Products on Stones by Portable NMR. *Gels* **7**(2021).
13. Guo, H., *et al.* Pro-Healing Zwitterionic Skin Sensor Enables Multi-Indicator Distinction and Continuous Real-Time Monitoring. *Adv. Funct. Mater.* (2021).
14. Diao, W., *et al.* Highly stretchable, ionic conductive and self-recoverable zwitterionic polyelectrolyte-based hydrogels by introducing multiple supramolecular sacrificial bonds in double network. *J. Appl. Polym. Sci.* **136**(2019).
15. Chen, Y., Li, J., Lu, J., Ding, M. & Chen, Y. Synthesis and properties of Poly(vinyl alcohol) hydrogels with high strength and toughness. *Polym. Test.* **108**(2022).
16. Hassan, C.M. & Peppas, N.A. Structure and Morphology of Freeze/Thawed PVA Hydrogels. *Macromolecules* **33**, 2472-2479 (2000).
17. Peppas, N.A. & Merrill, E.W. Differential scanning calorimetry of crystallized PVA hydrogels. *J. Appl. Polym. Sci.* **20**, 1457-1465 (1976).
18. Gupta, S., *et al.* Composition dependent structural modulations in transparent poly(vinyl alcohol) hydrogels. *Colloids Surf B Biointerfaces* **74**, 186-190 (2009).
19. Zhang, X.Z., Wu, D.Q. & Chu, C.C. Synthesis, characterization and controlled drug release of thermosensitive IPN-PNIPAAm hydrogels. *Biomaterials* **25**, 3793-3805 (2004).
20. Zhang, Z., *et al.* Double-network polyvinyl alcohol composite hydrogel with self-healing and low friction. *J. Appl. Polym. Sci.* **139**(2021).
