# Supplementary material for: Zwitterionic microgel preservation platform for circulating tumor cells in whole blood specimen
Source: Nat Commun. 2023 Aug 16;14:4958. doi: 10.1038/s41467-023-40668-1 (PMC10432405; doi:10.1038/s41467-023-40668-1)
Supplement: Supplementary file 2 — Description of additional supplementary files [file 41467_2023_40668_MOESM2_ESM.pdf]

Description of Additional Supplementary files

Supplementary Movie 1: The assembling process of ZBA and ZVA microgels

Supplementary Data 1: Detailed information of Regaent/Instrument of Molecular profiling analysis
