# Supplementary material for: Zwitterionic microgel preservation platform for circulating tumor cells in whole blood specimen
Source: Nat Commun. 2023 Aug 16;14:4958. doi: 10.1038/s41467-023-40668-1 (PMC10432405; doi:10.1038/s41467-023-40668-1)
Supplement: Supplementary file 5 — Reporting Summary [file 41467_2023_40668_MOESM5_ESM.pdf]

nature portfolio

Corresponding author(s): Prof. Jing Yang and Prof. Lei Zhang

Last updated by author(s): Yiming-Ma

Reporting Summary

Nature Portfolio wishes to improve the reproducibility of the work that we publish. This form provides structure for consistency and transparency in reporting. For further information on Nature Portfolio policies, see our Editorial Policies and the Editorial Policy Checklist.

Statistics

For all statistical analyses, confirm that the following items are present in the figure legend, table legend, main text, or Methods section.

n/a	Confirmed	
☐	☒	The exact sample size (*n*) for each experimental group/condition, given as a discrete number and unit of measurement
☐	☒	A statement on whether measurements were taken from distinct samples or whether the same sample was measured repeatedly
☐	☒	The statistical test(s) used AND whether they are one- or two-sided<br>*Only common tests should be described solely by name; describe more complex techniques in the Methods section.*
☒	☐	A description of all covariates tested
☐	☒	A description of any assumptions or corrections, such as tests of normality and adjustment for multiple comparisons
☐	☒	A full description of the statistical parameters including central tendency (e.g. means) or other basic estimates (e.g. regression coefficient) AND variation (e.g. standard deviation) or associated estimates of uncertainty (e.g. confidence intervals)
☐	☒	For null hypothesis testing, the test statistic (e.g. *F*, *t*, *r*) with confidence intervals, effect sizes, degrees of freedom and *P* value noted<br>*Give P values as exact values whenever suitable.*
☒	☐	For Bayesian analysis, information on the choice of priors and Markov chain Monte Carlo settings
☒	☐	For hierarchical and complex designs, identification of the appropriate level for tests and full reporting of outcomes
☐	☒	Estimates of effect sizes (e.g. Cohen's *d*, Pearson's *r*), indicating how they were calculated

Our web collection on statistics for biologists contains articles on many of the points above.

Software and code

Policy information about availability of computer code

Data collection	RNA integrity was assessed using the RNA Nano 6000 Assay Kit of the Bioanalyzer 2100 system (Agilent Technologies, CA, USA).<br>PCR products were purified (AMPure XP system) and library quality was assessed on the Agilent Bioanalyzer 2100 system<br>The clustering of the index-coded samples was performed on a cBot Cluster Generation System using TruSeq PE Cluster Kit v3-cBot-HS (Illumia) according to the manufacturer's instructions.
Data analysis	Pearson and Spearman correlation coefficients were used in correlation analysis by SPSS software (version 6.0)<br>Bar charts and curves were drawn by Graph Pad Prism (version 8.0.1)<br>Raw data (raw reads) of fastq format were firstly processed through in-house perl scripts<br>Reference genome and gene model annotation files were downloaded from genome website directly.<br>The mapped reads of each sample were assembled by StringTie (version 1.3.3b) (Mihaela Pertea.et al. 2015) in a reference-based approach<br>featureCounts v1.5.0-p3 was used to count the reads numbers mapped to each gene.<br>Differential expression analysis of two conditions/groups (two biological replicates per condition) was performed using the DESeq2 R package (version 1.20.0).<br>Gene Ontology (GO) enrichment analysis of differentially expressed genes was implemented by the clusterProfiler R package, in which gene length bias was corrected<br>At least 3 samples per group are needed per experiment to assess each statistical information<br>The results from Flow Cytometry tests were analyzed by FlowJo (version 10.6.2)

For manuscripts utilizing custom algorithms or software that are central to the research but not yet described in published literature, software must be made available to editors and reviewers. We strongly encourage code deposition in a community repository (e.g. GitHub). See the Nature Portfolio guidelines for submitting code & software for further information.

Data

Policy information about availability of data

All manuscripts must include a data availability statement. This statement should provide the following information, where applicable:

- Accession codes, unique identifiers, or web links for publicly available datasets
- A description of any restrictions on data availability
- For clinical datasets or third party data, please ensure that the statement adheres to our policy

All data are available in the main text and/or the supplementary materials.
All data generated or analyzed during this work are included in this submitted manuscript.
Source data are provided with this paper
The RNA-seq data that support the findings of this study are publicly accessible from the National Center for Biotechnology Information database.
The Biosample accession codes and links are:
SRR25208120
https://trace.ncbi.nlm.nih.gov/Traces/index.html?view=run_browser&acc=SRR25208120&display=metadata
SRR25208117
https://trace.ncbi.nlm.nih.gov/Traces/index.html?view=run_browser&acc=SRR25208117&display=metadata
SRR25208114
https://trace.ncbi.nlm.nih.gov/Traces/index.html?view=run_browser&acc=SRR25208114&display=metadata
SRR25208119
https://trace.ncbi.nlm.nih.gov/Traces/index.html?view=run_browser&acc=SRR25208119&display=metadata
SRR25208116
https://trace.ncbi.nlm.nih.gov/Traces/index.html?view=run_browser&acc=SRR25208116&display=metadata
SRR25208113
https://trace.ncbi.nlm.nih.gov/Traces/index.html?view=run_browser&acc=SRR25208113&display=metadata
SRR25208121
https://trace.ncbi.nlm.nih.gov/Traces/index.html?view=run_browser&acc=SRR25208121&display=metadata
SRR25208118
https://trace.ncbi.nlm.nih.gov/Traces/index.html?view=run_browser&acc=SRR25208118&display=metadata
SRR25208115
https://trace.ncbi.nlm.nih.gov/Traces/index.html?view=run_browser&acc=SRR25208115&display=metadata
SRR25208111
https://trace.ncbi.nlm.nih.gov/Traces/index.html?view=run_browser&acc=SRR25208111&display=metadata
SRR25208108
https://trace.ncbi.nlm.nih.gov/Traces/index.html?view=run_browser&acc=SRR25208108&display=metadata
SRR25208110
https://trace.ncbi.nlm.nih.gov/Traces/index.html?view=run_browser&acc=SRR25208110&display=metadata
SRR25208107
https://trace.ncbi.nlm.nih.gov/Traces/index.html?view=run_browser&acc=SRR25208107&display=metadata
SRR25208112
https://trace.ncbi.nlm.nih.gov/Traces/index.html?view=run_browser&acc=SRR25208112&display=metadata
SRR25208109
https://trace.ncbi.nlm.nih.gov/Traces/index.html?view=run_browser&acc=SRR25208109&display=metadata

Human research participants

Policy information about studies involving human research participants and Sex and Gender in Research.

Reporting on sex and gender	All participants are female
Population characteristics	A table of the exact patient characteristics is provided in Table 1 of the manuscript.
Recruitment	Whole blood samples were collected by Tianjin Medical University Cancer Institute and Hospital. The samples were used within 2 hours after collection and did not need to be stored.
Ethics oversight	Ethic Approval Statement<br>The Medical Ethic Committee of Tianjin Medical University Cancer Institute and Hospital conducted a serious rapid review of the above-mentioned research project. After review, the Medical Ethics Committee approved the protocol (protocol number is bc2021066) and agreed to the study “Zwitterionic Microgel Preservation Platform for Circulating Tumor Cells in Whole Blood Specimen” for research publication. Human Samples and Data usage in this research follow Privacy Protection Principle, which effectively protect personal privacy.

Note that full information on the approval of the study protocol must also be provided in the manuscript.

Field-specific reporting

Please select the one below that is the best fit for your research. If you are not sure, read the appropriate sections before making your selection.

☒ Life sciences ☐ Behavioural & social sciences ☐ Ecological, evolutionary & environmental sciences

For a reference copy of the document with all sections, see nature.com/documents/nr-reporting-summary-flat.pdf

Life sciences study design

All studies must disclose on these points even when the disclosure is negative.

Sample size	At least 3 samples per group are needed per experiment to assess statistical significance in pairwise comparisons. Data analysis primarily involves comparison of quantitative endpoints between experimental groups. In such settings, the number of samples to be used in each experiment may vary, depending on the variability of the endpoint relative to what is considered a biologically meaningful difference. For example, if the standard deviation of the outcome is 50% of the biologically meaningful difference between groups (or equivalently, the 'effect size' is 2.0 SD units) then 3 cells/blood samples per group is sufficient. In experiments where the outcome is relatively more variable, up to 20 samples per group may be needed.
Data exclusions	Technically validated results were always included to the analyses and we did not apply any exclusion criteria for outliers.
Replication	All experiments were conducted for at least three independent times to justify good reproducibility. All attempts at replication were successful.
Randomization	All replications done in randomized order on different experiments. The researchers did not subjectively select starting materials or cells.
Blinding	Examination and quantification were done by three independent researchers blinded to sample identity. Materials synthesis, cell preservation, flow cytometry, and RNA-seq work were done by different researchers.

Reporting for specific materials, systems and methods

We require information from authors about some types of materials, experimental systems and methods used in many studies. Here, indicate whether each material, system or method listed is relevant to your study. If you are not sure if a list item applies to your research, read the appropriate section before selecting a response.

Materials & experimental systems

n/a	Involved in the study
☐	☒ Antibodies
☒	☐ Eukaryotic cell lines
☒	☐ Palaeontology and archaeology
☒	☐ Animals and other organisms
☒	☐ Clinical data
☒	☐ Dual use research of concern

Methods

n/a	Involved in the study
☒	☐ ChIP-seq
☐	☒ Flow cytometry
☒	☐ MRI-based neuroimaging

Antibodies

Antibodies used	For each antibodies we described --> name/manufacturer /clone /cat.number/dilution<br>CD45 Monoclonal Antibody APC/Cyanine7/Biolegend/2D1/368516/ 5 µl antibodies per 10^6 cells in 100 µl staining volume<br>CD62P (P-Selectin) Monoclonal Antibody PE/Invitrogen/Psel.KO2.3/12-0626-82/5 µl antibodies per 10^6 cells in 100 µl staining volume<br>CD326 (EpCAM) Monoclonal Antibody Alexa Fluor 488/Invitrogen/MH99/53-8326-42/5 µl antibodies per 10^6 cells in 100 µl staining volume
Validation	All the antibodies were made from Biolegend and Invitrogen and they were well characterized and validated by providers.<br><br>Biolegend quality control and validation(the detailed information could be found in https://www.biolegend.com/Files/Images/BioLegend/literature/images/07-0139-02_IHC.pdf):<br>The specificity and sensitivity of each antibody is thoroughly validated in the New Product Development stage. This is done by staining multiple target cells with either single- or multi-color analysis or by other testing approaches. The QC specifications and testing SOPs and gold standard for each product are then developed.<br>The functional performance of each batch of BioLegend products is strictly QC-tested according to the established QC procedures. In general, each product is tested using the following criteria:<br>Staining of 1-3 target cell types with either single- or multi-color analysis detailed in the QC specification (including positive and negative controls). The tested cells can be primary cells and/or cell lines known to be positive or negative for the target antigen.

Each batch product is validated by QC testing with a series of dilutions to make sure the product is working within expected antibody titer range.
Each batch is compared to an internally established "gold standard" to maintain batch-to-batch consistency.
When applicable, our products are side-by-side tested with our competitors' products to make sure that BioLegend's products exceed or are at least the same quality.
For most tandem dye-conjugated products, color compensation is examined in order to verify tandem integrity.
Invitrogen statement: Antibodies are some of the most critical research reagents used in the lab. To help ensure superior antibody results, we've expanded our specificity testing methodology using a two-part approach for advanced verification.

Invitrogen quality control and validation(the detailed information could be found in https://www.thermofisher.cn/cn/en/home/life-science/antibodies/invitrogen-antibody-validation.html):
Antibodies are some of the most critical research reagents used in the lab. Poor specificity or application performance can significantly frustrate the ability to obtain good results, which can cause critical delays. Underperforming antibodies result in a lack of reproducibility, wasting time and money. In other words, researchers need antibodies that bind to the right target and work in their applications every time. To help ensure superior antibody results, we've expanded our specificity testing methodology using a two-part approach for advanced verification.
Invitrogen antibodies are currently undergoing a rigorous two-part testing approach
Part 1—Target specificity verification:
This helps ensure the antibody will bind to the correct target. Our antibodies are being tested using at least one of the following methods to ensure proper functionality in researcher's experiments. Click on each testing method below for detailed testing strategies, workflow examples, and data figure legends.
Knockout—expression testing using CRISPR-Cas9 cell models
Knockdown—expression testing using RNAi to knockdown gene of interest
Independent antibody verification (IAV)—measurement of target expression is performed using two differentially raised antibodies recognizing the same protein target
Cell treatment—detecting downstream events following cell treatment
Relative expression—using naturally occurring variable expression to confirm specificity
Neutralization—functional blocking of protein activity by antibody binding
Peptide array—using arrays to test reactivity against known protein modifications
SNAP-ChIP™—using SNAP-ChIP to test reactivity against known protein modifications
Immunoprecipitation-Mass Spectrometry (IP-MS)—testing using immunoprecipitation followed by mass spectrometry to identify antibody targets
Part 2—Functional application validation
These tests help ensure the antibody works in a particular application(s) of interest, which may include
(but are not limited to):
Western blotting
Flow cytometry
ChIP
Immunofluorescence imaging
Immunohistochemistry
Most antibodies were developed with specific applications in mind. Testing that an antibody generates acceptable results in a specific application is the second part of confirming antibody performance.

Flow Cytometry

Plots

Confirm that:

☒ The axis labels state the marker and fluorochrome used (e.g. CD4-FITC).

☒ The axis scales are clearly visible. Include numbers along axes only for bottom left plot of group (a 'group' is an analysis of identical markers).

☒ All plots are contour plots with outliers or pseudocolor plots.

☒ A numerical value for number of cells or percentage (with statistics) is provided.

Methodology

Sample preparation

Preparation of model CTCs: 2000 of MCF-7 cells were added in 1 mL whole blood to simulate CTCs. Blood samples were extracted from different breast cancer patients.
Hypothermic preservation of whole blood with model CTCs: To preserve model CTCs in whole blood samples, ZBVA hydrogel system was established by adding 33 mg of ZBA and ZVA microgels into whole blood samples. The CPDA solution system was obtained by mixing 1 mL of blood sample with 150 µL of CPDA. All preservation systems were then straightly stored in a refrigerator.
Viability and apoptosis test of model CTCs: After separtion from ZBVA and CPDA system, model CTCs in the whole blood were then enriched by CTCs EasySep kit with magnetic activated cell sorting method. To accurately detect model CTC viability and distinguish them from white blood cells, residual red blood cells, and platelets, the enriched solutions were stained with CD45-APC, Annexin-V-FITC, and 7AAD, and gently homogenized in a vortex mixer. After a 30-minute incubation at room temperature, the stained solution was analyzed using the FASCAria III flow cytometer (BD Bioscience, USA).

Instrument

Cell events were collected with an FASCAria III flow cytometer (BD Bioscience, USA)

Software	Flow cytometry data was collected with BD FACS software (BD Biosciences) and analyzed with FlowJo software (TreeStar, Ashland, OR)
Cell population abundance	No sorting was performed with the flow cytometer
Gating strategy	For fresh cells, gating strategy to separate cell events from debris using forward scatter vs. side scatter plot is described in the first row of Supplementary Figure 24. Gating strategy for Figure 4b and Supplementary Figure 16 are described in Supplementary Figure 24. Fluorescently labeled CD326+ but CD45- represent model CTC cells. Fluorescently Annexin V- and 7AAD- represent the living cells.

☒ Tick this box to confirm that a figure exemplifying the gating strategy is provided in the Supplementary Information.
